# Screen Media Use and Mental Health of Children and Adolescents: A Secondary Analysis of a Randomized Clinical Trial

**DOI:** 10.1001/jamanetworkopen.2024.19881

**Published:** 2024-07-12

**Authors:** Jesper Schmidt-Persson, Martin Gillies Banke Rasmussen, Sarah Overgaard Sørensen, Sofie Rath Mortensen, Line Grønholt Olesen, Søren Brage, Peter Lund Kristensen, Niels Bilenberg, Anders Grøntved

**Affiliations:** 1 Department of Sports Science and Clinical Biomechanics, Research Unit for Exercise Epidemiology, Centre of Research in Childhood Health, University of Southern Denmark, Odense, Denmark; 2 Applied Research in Child and Adult Health, Department of Midwifery, Physiotherapy, Occupational Therapy, and Psychomotor Therapy, University College Copenhagen, Copenhagen, Denmark; 3 Steno Diabetes Center Odense, Odense University Hospital, Odense, Denmark; 4 Research and Implementation Unit PROgrez, Department of Physiotherapy and Occupational Therapy, Næstved-Slagelse-Ringsted Hospitals, Region Zealand, Denmark; 5 Steno Diabetes Center Aarhus, Aarhus University Hospital, Skejby, Aarhus, Denmark; 6 MRC Epidemiology Unit, University of Cambridge, Cambridge, United Kingdom; 7 Child and Adolescent Psychiatric Department, Mental Health Hospital and University Clinic, Region of Southern Denmark, Odense, Denmark

## Abstract

**Importance:**

Excessive screen media use has been associated with poorer mental health among children and adolescents in several observational studies. However, experimental evidence supporting this hypothesis is lacking.

**Objective:**

To investigate the effects of a 2-week screen media reduction intervention on children’s and adolescents’ mental health.

**Design, Setting, and Participants:**

This prespecified secondary analysis of a cluster randomized clinical trial with a 2-week follow-up included 89 families (with 181 children and adolescents) from 10 Danish municipalities in the region of Southern Denmark. All study procedures were carried out in the home of the participants. Enrollment began on June 6, 2019, and ended on March 30, 2021. This analysis was conducted between January 1 and November 30, 2023.

**Intervention:**

Families were randomly allocated to a screen media reduction group or a control group. The 2-week screen media reduction intervention was designed to ensure a high level of compliance to the reduction in leisure-time screen media use. Participants allocated to the intervention group had to reduce their leisure-time screen media use to 3 hours per week or less per person and hand over smartphones and tablets.

**Main Outcomes and Measures:**

The main outcome was the between-group mean difference in change in total behavioral difficulties, measured by the Strengths and Difficulties Questionnaire at 2-week follow-up. Results were estimated using mixed-effects tobit regression models. Analyses were carried out as both intention to treat and complete case.

**Results:**

In the sample of 89 families including 181 children and adolescents (intervention group [45 families]: 86 children; mean [SD] age, 8.6 [2.7] years; 42 girls [49%]; control group [44 families]: 95 children; mean [SD] age, 9.5 [2.5] years; 57 girls [60%]), there was a statistically significant between-group mean difference in the total difficulties score, favoring the screen media reduction intervention (−1.67; 95% CI, −2.68 to −0.67; Cohen *d*, 0.53). The greatest improvements were observed for internalizing symptoms (emotional symptoms and peer problems; between-group mean difference, −1.03; 95% CI, −1.76 to −0.29) and prosocial behavior (between-group mean difference, 0.84; 95% CI, 0.39-1.30).

**Conclusions and Relevance:**

This secondary analysis of a randomized clinical trial found that a short-term reduction in leisure-time screen media use within families positively affected psychological symptoms of children and adolescents, particularly by mitigating internalizing behavioral issues and enhancing prosocial behavior. More research is needed to confirm whether these effects are sustainable in the long term.

**Trial Registration:**

ClinicalTrials.gov Identifier: NCT04098913

## Key Points

**Question:**

Does reducing leisure-time screen media use improve mental health among children and adolescents?

**Findings:**

In this prespecified secondary analysis of a randomized clinical trial including 89 families (181 children and adolescents), reducing screen media use had an overall positive effect on children’s and adolescents’ behavioral difficulties. The most noticeable benefits associated with reduced screen media use was a decrease in internalizing behavioral issues and enhanced positive social interactions.

**Meaning:**

The findings provide evidence for a causal link between a short-term reduction in screen media use during leisure and improvements in children’s and adolescents’ psychological symptoms.

## Teaser

This secondary analysis of a randomized clinical trial investigates the effects of a 2-week leisure-time screen media reduction intervention on the mental health of children and adolescents.

## Introduction

The proportion of children and adolescents with poor mental health has increased over the past years in many countries.^1,2,3,4^ In the US, the number of adolescents reporting poor mental health during the past month was 29% in the 2021 Youth Risk Behavior Survey.^5^ Based on data from 45 European countries, 25% of 11- to 15-year-old adolescents reported symptoms related to psychological health (such as nervousness, irritability, and difficulties falling asleep).^6^ Individuals are particularly vulnerable to negative or stressful environmental experiences during adolescence.^7^ Furthermore, research shows that self-reported life satisfaction has the steepest decrease during adolescence compared with other stages of the lifespan.^8^

The past decades have brought substantial advancements in digital technology, and more children and adolescents have their own personal screen media devices.^9,10,11^ Use of screen media devices has become a central aspect in children’s and adolescents’ daily lives, offering endless options for entertainment including watching videos, gaming, social media interaction, and communicating with family and friends.^9,10,11^ Screen media use is a complex and broad construct that includes active and passive engagement with different types of devices and content.^12^ Although the use of screen media devices, particularly smartphones, can facilitate many daily activities and some social interactions, concerns have been raised over the past years about the potential negative effect of digital screen media use on children’s and adolescents’ mental health,^13^ yet the research is still sparse and remains inconclusive.^14,15,16,17,18,19,20^ Some observational studies find moderate-sized associations between high levels of screen media use and poor mental health,^15,21^ while some researchers claim that observed associations are too weak to have any societal relevance.^17^ A recent systematic review and meta-analysis of 87 studies on the association between children’s screen media use and internalizing and externalizing symptoms found small but significant positive associations, suggesting that higher levels of screen media use might be associated with more behavioral problems.^22^ However, all the included studies were observational and based mainly on self-reported measures of screen media use. Thus, carefully designed randomized clinical trials are warranted to investigate the potential short-term and long-term effects of reducing screen media use on children’s and adolescents’ mental health. The present study focuses on the short term and investigates the effect of a family-based, 2-week break from leisure-time screen media use on children’s and adolescents’ mental health.

## Methods

### Study Design

This study is a prespecified secondary analysis of the SCREENS (Short-term Efficacy of Reducing Screen-Based Media Use) trial, which is a parallel cluster randomized clinical trial (study protocol available in Supplement 1).^23^ Results for the primary outcome of the SCREENS trial and a secondary analysis have previously been reported.^24,25^ The primary outcome analysis revealed that children and adolescents allocated to the screen media reduction group increased their nonsedentary leisure time by 45 minutes per day compared with children and adolescents in the control group.^24^ In the present study, we report the results for children’s and adolescents’ behavioral strengths and difficulties based on data from the Strengths and Difficulties Questionnaire (SDQ).^26,27^ The SDQ has been found to be a useful measure of young people’s mental health.^27^ The trial design was developed on the basis of the results and experiences from the pilot trial.^28^ Family enrollment started on June 6, 2019, and the final follow-up assessments were completed on March 30, 2021. Ethical approval for the SCREENS trial was granted by the Ethical Committee of Southern Denmark. All families provided written informed consent before undergoing any assessments; parents provided consent verbally and in writing on behalf of their children, but children were present and could ask questions to their parents and the research staff. Signs of child dissent were considered contraindicatory to participation. This study is reported in compliance with the Consolidated Standards of Reporting Trials (CONSORT) reporting guideline and statement for cluster randomized trials.

### Participants

We recruited families from a population-based survey concerning family screen media habits.^11,29^ A survey invitation was sent to parents with at least 1 child aged 6 to 10 years in 10 municipalities in the region of Southern Denmark.^11^ Recipients of the survey were randomly selected by the Danish Health Data Authority using data from the Danish Civil Registration system. At the end of the survey, responders were asked to answer (yes or no) whether they were interested in receiving more information about another study: a family-based screen media reduction trial. We assessed families’ eligibility among respondents who answered yes. Initial eligibility criteria were that the respondent parent had to have leisure-time screen media use above the 40th percentile (based on the first 1000 responses) and have a full-time job or study full time. We excluded families with children younger than 4 years of age living in the household. A research team member telephoned the family if these criteria were fulfilled, to confirm that at least 1 child and 1 adult in the household wanted to participate and were able to hand over smartphones and tablets to the researchers. The study also included a measurement of physical activity using accelerometers. Therefore, participants were not eligible if they could not engage in regular physical activities of daily life, had a sleep disorder, or had any neuropsychiatric or developmental disorders.

### Randomization Procedure

The randomization was performed on day 8 of the trial after completion of baseline assessments in the families’ homes. A web-based randomization platform operated by a third party (Odense Patient Data Explorative Network) was used to perform the randomization. The Odense Patient Data Explorative Network generated the random sequence in blocks of 2 to 4 families and ensured allocation concealment until a family was assigned to a group. We chose to randomize and intervene on a family level rather than based on individuals to increase compliance with the intervention and decrease potential contamination between experimental conditions.

### Intervention

The aim of the trial was to compare a reduction in leisure-time screen media use (intervention) with usual leisure-time screen media use (control) in families with children. The intervention lasted for 2 weeks and consisted of the following elements. All children and at least 1 adult (preferably all adults in the household) had to hand over smartphones or tablets during the 2 weeks of the intervention. They were also asked to reduce their leisure-time screen media use (all use of screen media devices outside self-reported work and school hours) on other devices, such as televisions and computers, to 3 hours per week or less. Participants who handed over their smartphone received a non-smartphone in return that could call and send text messages. Participants were asked to register all leisure-time screen media use during the intervention. Small intervention reminder posters were put up in the household, and each family received a small financial reimbursement of approximately €70 (US $76). Participants were allowed 30 minutes per day of necessary screen media use to enable coordination of appointments, completion of school assignments, and other necessary tasks. Necessary screen media use was not considered a part of their leisure-time screen media use. A more detailed description of the intervention and the theoretical framework underpinning the intervention is described in the study protocol.^23^ Families allocated to the control group were asked to carry on with their usual screen media habits until they completed the follow-up measurements, at which time they would be offered to try the intervention condition.

### Intervention Compliance

As outlined in the previous study,^24^ we evaluated adherence to the screen media reduction intervention using objective measures, including research-based smartphone and tablet tracker applications,^30^ PC software, and television monitors. In the screen media use reduction group, 97% of the children and adolescents (83 of 86) were sufficiently compliant to the intervention, defined a priori as having less than or equal to 7 hours per week of leisure-time screen media use to ensure a distinct contrast with the control group, while allowing for a marginal deviation from the target.^24^

### Outcomes

We administrated the SDQ as part of a larger questionnaire at both baseline and follow-up to assess changes in children’s and adolescents’ behavioral strengths and difficulties. The SDQ has been found to be valid and suitable for use in randomized clinical trials.^31,32^ Parents answered the questionnaire on behalf of their children at the start of the baseline protocol before randomization and at follow-up (at the end of the intervention), with 21 days between assessments. At baseline, parents were asked to report based on the previous 6 months, and at follow-up, they were asked to report based on the past 2 weeks. A digital link to an age-appropriate Danish version of the SDQ (versions: 2-4 years, 5-6 years [not in school], 4-10 years [in school], and 11-17 years) was sent to the parents via email. The SDQ consists of 25 items (eg, “Considerate of other people’s feelings”) for which parents have to answer 1 of the following 3 categories: not true (scored as 0), somewhat true (scored as 1), and certainly true (scored as 2). Five items were scored in the reverse order (ie, not true [scored as 2], somewhat true [scored as 1], and certainly true [scored as 0]). Items were then summarized into 5 different subscales (ie, emotional symptoms, conduct problems, hyperactivity or inattention, peer problems, and prosocial behavior). A higher score denotes more behavioral difficulties, except in the case of the prosocial behavior score, in which a higher score indicates favorable behavior. Externalizing symptoms (score range, 0-20 points) were calculated as the sum of the conduct problems and the hyperactivity or inattention scales. Internalizing symptoms (score range, 0-20 points) were calculated as the sum of the emotional symptoms and peer problems scales. Total difficulties (score range, 0-40 points) were generated by summing all subscales except for the prosocial behavior scale.

### Statistical Analysis

Statistical analysis was conducted between January 1 and November 30, 2023. We constructed violin plots to depict the distribution for the SDQ scores for total difficulties, internalizing symptoms, and externalizing symptoms among both groups at baseline. Mixed-effects tobit regression models were used to estimate the between-group mean differences in the total difficulties score, externalizing score, internalizing score, emotional symptoms, conduct problems, hyperactivity or inattention, peer problems, and prosocial behavior, with family ID as a random effect to account for the clustered design of the study. Tobit regression was used to account for potential floor effects,^33^ with values censored at 0 (lower limit) because scores were low at baseline among many participants. All models were adjusted for age because there was a slight imbalance between groups at baseline. Analyses were carried out as intention to treat, and 95% CIs were estimated. We report complete-case analyses for all outcomes, and for the primary analysis (change in total difficulties score), we also report a sensitivity analysis in which missing change scores were imputed for 20 of 181 participants (11%) with missing SDQ data at baseline and/or follow-up using multiple imputation by chained equations with 20 imputed datasets.^34^ The imputation model included biological sex, age, total leisure-time screen media use at baseline, parental educational level, and child’s body mass index *z* score. We also conducted post hoc subgroup analyses for the total difficulties score, examining statistical interactions based on biological sex, age groups, and baseline amount of leisure-time screen media use. We examined assumptions of mixed-effects tobit regression models and found no violations. Statistical analyses were carried out using Stata, version 18 (StataCorp LLC). All *P* values were from 2-sided tests, and results were deemed statistically significant at *P* < .05. A per-protocol analysis was not carried out due to near perfect compliance.

## Results

The flow of study participants has been described in detail elsewhere^24^ and is presented in the eFigure in Supplement 2. In summary, we found 408 eligible families among 1420 interested families identified from a population-based survey concerning screen media behaviors in families with children.^11^ A total of 92 families agreed to participate in the trial and completed baseline assessments. Three families dropped out after baseline assessments, and 89 families (including a total of 181 children) were randomized to either the screen media reduction group (45 families [86 children]: mean [SD] age, 8.6 [2.7] years; 42 girls [49%] and 44 boys [51%]) or the control group (44 families [95 children]: mean [SD] age, 9.5 [2.5] years; 57 girls [60%] and 38 boys [40%]) (Table). Baseline characteristics were generally similar across the 2 groups.

**Table.** Baseline Characteristics^a^

Characteristic	Control (n = 44 families)	Intervention (n = 45 families)
Children, No.	95	86
Age, mean (SD) [range], y	9.5 (2.5) [4-15]	8.6 (2.7) [4-17]
Sex, No./total No. (%)		
Female	57/95 (60)	42/86 (49)
Male	38/95 (40)	44/86 (51)
BMI *z* score, mean (SD)	0.1 (1.2)	0.2 (1.0)
Leisure-time smartphone or tablet use, median (IQR), h/wk	9.6 (3.1-15.9)	10.4 (3.6-19.7)
Leisure-time computer use, median (IQR), h/wk	10.1 (6.0-19.7)	9.4 (2.1-27.0)
Leisure-time television use, median (IQR), h/wk	4.7 (1.5-10.2)	6.2 (2.4-9.6)
Adults, No.	82	82
Educational attainment, No./total No. (%)^b^		
ISCED 0-3	16/82 (20)	14/82 (17)
ISCED 4-6	40/82 (49)	48/82 (59)
ISCED 7-8	26/82 (32)	20/82 (24)
Household environment		
No. of children, median (range)	2 (1-4)	2 (1-4)
No. of adults, median (range)	2.0 (1-3)	2.0 (1-3)
No. of devices, median (IQR)	11.0 (8.0-12.0)	9.0 (7.0-11.0)

Abbreviations: BMI, body mass index (calculated as weight in kilograms divided by height in meters squared); ISCED, International Standard Classification of Education.

^a^ Baseline characteristics of included children, their parents’ educational attainment according to the ISCED, and the household environment.

^b^ ISCED scores: 0 to 3, early childhood education to upper secondary education or equivalent; 4 to 6, postsecondary nontertiary education to bachelor’s degree or equivalent; and 7 to 8, master’s degree to doctorate or equivalent.

**Figure 1.**
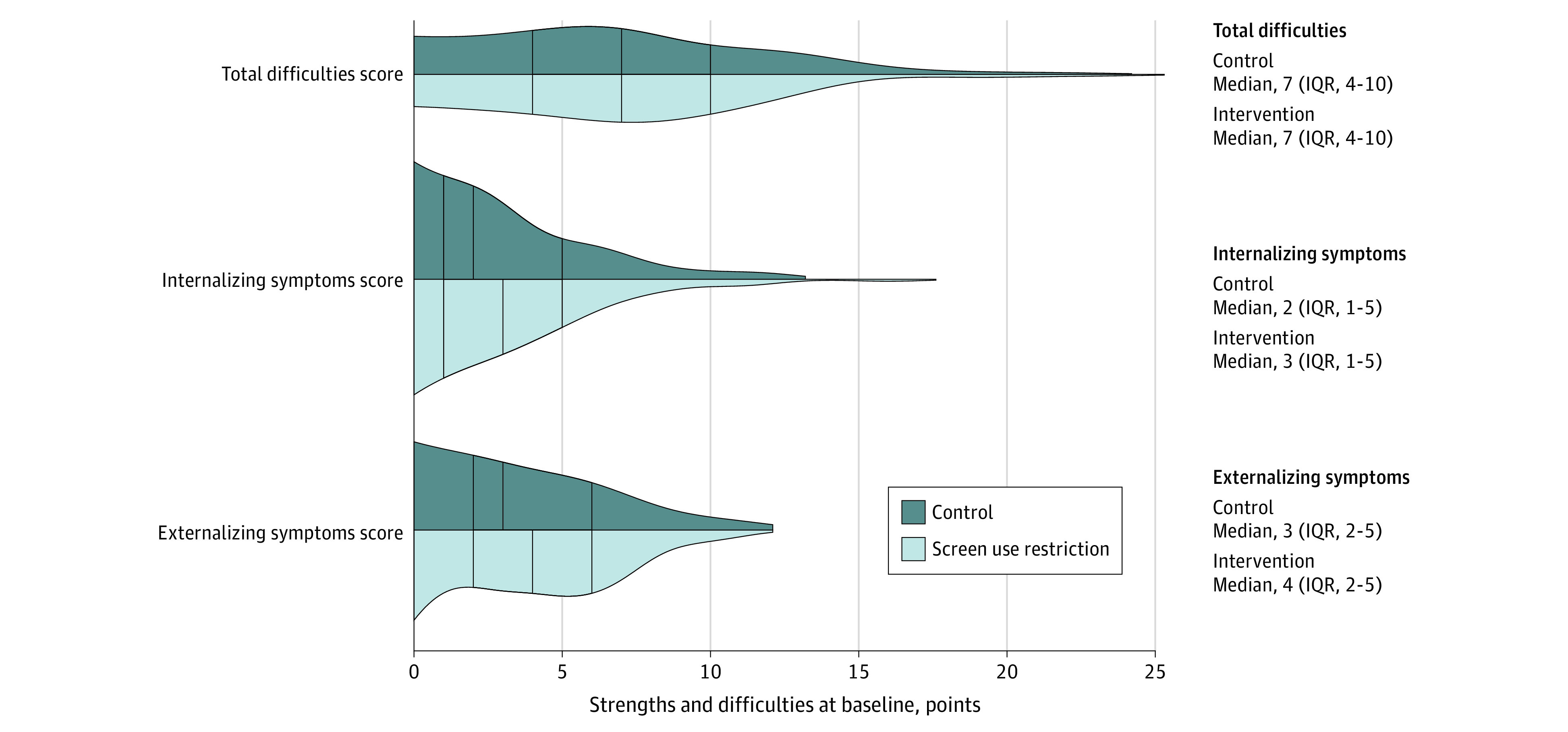
Violin Plots of Total Difficulties, Internalizing Symptoms, and Externalizing Symptoms Scores Violin plots display the estimated density of the scores derived from the Strengths and Difficulties Questionnaire data by group at baseline. The median and IQRs are shown within the plots for each group (vertical lines).

The violin plots (Figure 1) illustrate that the distributions of the total difficulties scores, internalizing symptoms scores, and externalizing symptoms scores were similar between groups at baseline. A substantial number of participants registered a baseline value of zero for each of these outcomes. Moreover, most children’s scores fell within the normal range for Danish children aged 5 to 17 years.^35,36^

A total of 8 participants (4%) had missing SDQ data at baseline, and 19 participants (11%) had missing SDQ data at follow-up. Of those who had missing SDQ data at follow-up, 9 participants were from the screen media reduction group, and 10 participants were from the control group. Participants with missing SDQ data at follow-up had a mean (SD) age of 9.3 (3.0) years, and 67% (8 of 12) were girls (eTable 1 in Supplement 2). Total difficulties scores were similar for participants with missing data at follow-up compared with those with complete data at baseline and follow-up.

### Effects of the Screen Media Reduction Intervention on Mental Health

**Figure 2.**
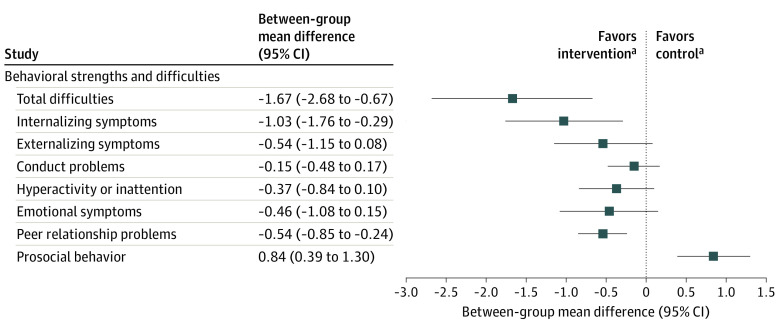
Between-Group Mean Difference in Change in Behavioral Strengths and Difficulties From Baseline to Follow-Up Estimates are from mixed-effects tobit regression models adjusted for age (n = 174). Internalizing symptoms, emotional and peer problems; externalizing symptoms, conduct and hyperactivity problems. ^a^Negative values favor the intervention and positive values favor the control, with the exception of prosocial behavior, for which this relationship is reversed.

The effects of the screen media reduction intervention on children’s behavioral strengths and difficulties are presented in Figure 2 and eTable 2 in Supplement 2. There was a statistically significant between-group mean difference in change on the total difficulties score from baseline to follow-up of −1.67 (95% CI, −2.68 to −0.67) in favor of the screen media reduction intervention, which corresponds to a Cohen *d* effect size of 0.53. The sensitivity analysis for the total difficulties score with missing data imputed using chained equations yielded similar results, with a between-group mean difference of −1.71 (95% CI, −3.10 to −0.33). Analyses of the SDQ subscales (internalizing and externalizing symptoms) showed that the effect was most pronounced on children’s internalizing symptoms (ie, emotional problems and peer problems; −1.03; 95% CI, −1.76 to −0.29). The analysis of the score for the prosocial subscale showed a significant intervention effect in favor of the screen media reduction group (0.84; 95% CI, 0.39-1.30; Figure 2). Removing the adjustment for age did not alter the results in any of the analyses.

### Subgroup Analyses for the Total Difficulties Score

**Figure 3.**
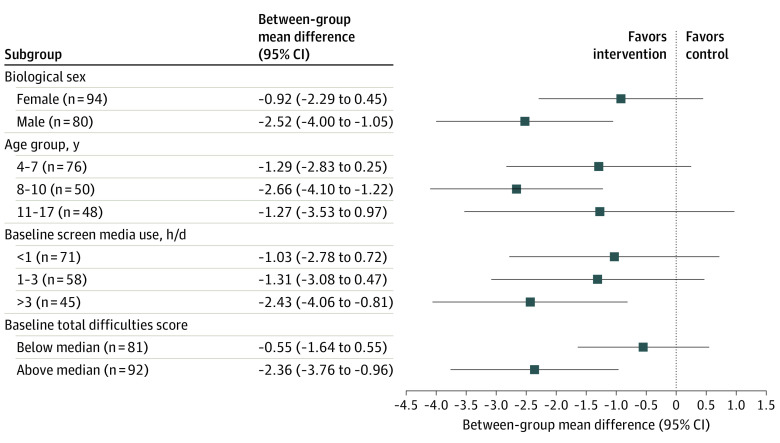
Subgroup Analyses From Baseline to Follow-Up The subgroup analyses show subgroup-level estimates from mixed-effects tobit regression models. The median score for baseline total difficulties was 7 (below median: <7, median or above: ≥7). Analyses were adjusted for age, except for the age group analysis.

The results of the post hoc subgroup analyses are presented in Figure 3. We found no statistically significant interactions in the subgroup analyses performed. However, the point estimates were higher for boys, children with higher screen media use at baseline, and children with higher total difficulties at baseline.

## Discussion

Results from this cluster randomized clinical trial showed that reducing leisure-time screen media use in families for a period of 2 weeks had a significant positive effect on children’s and adolescents’ mental health. Considering the different mental subdomains, a reduction in screen media use resulted in improvements in internalizing symptoms (emotional symptoms and peer problems) and prosocial behavior. This finding suggests that while limiting screen media use had a broad positive effect on behavioral difficulties, the most pronounced benefits associated with limiting screen media use may be in mitigating internalizing behavioral issues and enhancing positive social interactions among children and adolescents.

To our knowledge, this is the first family-based randomized clinical trial examining the effect of reducing leisure-time screen media use on children’s and adolescents’ mental health. Our study confirms that reducing leisure-time screen media use improves several aspects of mental health among children and adolescents in the short term, which is in line with results of several observational studies showing that higher levels of screen media use are associated with poorer mental health among children and adolescents.^22,37^ In contrast to previous studies reporting that effect sizes on the association between screen media use and mental health were negligible,^17^ the results of our trial suggest that the effect size is moderate (Cohen *d* = 0.53). The observed mean difference of 1.67 points in the SDQ total difficulties score mirrors effects seen in multifocus treatments for mental health conditions among youths.^38,39^ When extrapolated to the Danish population of children, a downward shift in total difficulties score corresponding to the effect sizes seen among boys and girls would notably alter the distribution within the SDQ’s diagnostic categories, particularly decreasing the proportion classified as abnormal for both boys and girls (eAppendix and eTable 3 in Supplement 2). However, the intervention is not designed to be implemented among families in the long term, which, in combination with the short-term follow-up of 2 weeks, limits the generalizability of the results to families’ everyday lives. However, the results highlight that positive mental health effects can be achieved by taking a short break from screen media use as a family.

There were some indications that boys, compared with girls, and that children aged 8 to 10 years, compared with those aged 4 to 7 years and 11 to 17 years might respond better to the screen reduction intervention. This difference in response might be caused by different types of engagement with screen media devices and use of different screen media content across sex and age groups,^9,10,11^ which could affect mental health differently. Also, the results suggest that the intervention may have a greater effect among children and adolescents with higher levels of screen media use and higher total difficulties scores at baseline. However, these subgroup differences were not statistically significant. Given that our study sample was drawn from a general population of children and adolescents, rather than from a high-risk group, a large portion of the participants reported minimal behavioral difficulties. This left limited room for improvement but stresses the need for further trials in high-risk groups. In the analysis stratified by baseline level of the total difficulties score, a more pronounced favorable effect of the intervention was observed among individuals scoring above the median for total difficulties. In contrast, we observed no significant intervention effect among those scoring below the median for total difficulties score at baseline. However, future studies are needed to investigate whether reducing screen media use has a greater effect among groups with higher total difficulties scores.

We can only speculate about the mechanisms underlying the positive effects of limiting screen media use on children’s and adolescents’ mental health. When children and adolescents spend much of their leisure time using screen media devices, a putative effect may be diminishing face-to-face social engagement with friends, peers, and family members.^40,41^ A possible explanation of the positive effects found among both children and adolescents could be that parents participating in the SCREENS trial also reported improved mental well-being (WHO-5 Well-being Index score), as reported previously.^25^ The positive effects may also be explained by increases in shared leisure time among family members without use of screen media devices during the intervention, potentially making room for more social interactions. Reduced interpersonal engagement could heighten emotional symptoms, manifesting as amplified feelings of isolation, loneliness, and social anxiety.^42,43^ Beyond the sheer volume of screen time and the potential displacement of face-to-face interactions, the specific type and content of digital engagements, especially on platforms such as social media, might play a critical role in influencing mental well-being. Randomized experiments with young or middle-aged adults indicate that limiting social media use yields positive psychological outcomes across various age groups. For instance, among college-aged individuals, short daily limits or brief breaks from social media have been associated with decreased feelings of loneliness and depression symptoms.^44^ Similarly, among adults, either taking a short break from social media or setting strict daily limits has been associated with improved well-being and reduced symptoms of depression and anxiety.^45,46^ Also, prospective observational studies suggest that reduced and intentional social media use is associated with better mood and mental well-being among young individuals.^47^ Results from a recent observational study indicate that children and adolescents may be more sensitive to negative effects of social media during specific periods (ie, males aged 14-15 years and 19 years and females aged 11-13 years and 19 years).^48^ We did not observe any consistent age-dependent effects of reducing general leisure screen media use; however, further studies are needed to investigate this.

### Strengths and Limitations

This study has some strengths. A key strength is its design as an efficacy study with high ecological validity. Our cluster randomized clinical trial, conducted in young people’s natural environments, allows for a more rigorous examination of causality compared with investigations in observational studies. In addition, objective assessment of compliance to the screen media reduction intervention further enhances the internal validity of our findings, ensuring that participants genuinely reduced their screen media use. Another strength is the low dropout and missing data rate, which minimizes the potential for attrition bias.

Nevertheless, the findings must be interpreted with some limitations in mind. First, the open-label nature of the study might have introduced bias in the assessment of behavioral strengths and difficulties, as the parents’ responses could have been influenced by their knowledge of group allocation. Second, some families in the control group also decreased their leisure-time screen media use to a moderate extent and thus reduced the contrast in screen media use between the 2 conditions, which may have resulted in an underestimation of the intervention effect. Third, while parental involvement was crucial for achieving a high level of compliance with the intervention, parents, children, and adolescents in this study may represent a subgroup with a particularly high motivation to reduce screen media use, as families volunteered for participation, which could influence the generalizability of the results. Fourth, the use of the parented-reported version of the SDQ reflects the parent’s perception of the child’s behavioral strengths and difficulties to a larger degree, rather than the child’s own perception.

## Conclusions

Taken together, the results of this secondary analysis of a randomized clinical trial show that when entire families—both parents, children, and adolescents—reduce their leisure-time screen media use for 2 weeks, it can positively affect children’s and adolescents’ behavioral strengths and difficulties. The intervention targeted an overall reduction in screen media use during leisure time, without pinpointing specific types of screen media activities. Future research should explore the potential differential effects of various types of screen media use and look deeper into whether collective family participation in such interventions is a pivotal component for observed benefits. Moreover, more research is needed to confirm whether these effects are sustainable in the long term.

## Notes

Supplement 1.
Trial Protocol

Supplement 2. **eFigure.** Flow Chart **eTable 1.** Comparison of Total Difficulties Scores at Baseline **eTable 2.** Analyses for Behavioral Strengths and Difficulties **eTable 3.** Comparison of Norm Data Proportions to Simulated Proportions
eAppendix.

Supplement 3.
Data Sharing Statement
