# Supplementary material for: Screen Media Use and Mental Health of Children and Adolescents: A Secondary Analysis of a Randomized Clinical Trial
Source: JAMA Netw Open. 2024 Jul 12;7(7):e2419881. doi: 10.1001/jamanetworkopen.2024.19881 (PMC11245724; doi:10.1001/jamanetworkopen.2024.19881)
Supplement: Supplement 1. — Trial Protocol [file jamanetwopen-e2419881-s001.pdf]

Supplementary material: Study protocol

Manuscript: Effects of reducing screen media use on mental health in children and adolescents

This file contains the following items:

I. Original study protocol (1st Ethics approved study protocol) [25.02.2019]

II. Final study protocol [10.12.2019]

III. Study protocol revision sequence and summary of changes

Original study protocol (1st Ethics approved study protocol)

Title: The acute effect of reduced or restricted screen-time on physical activity, physiological stress, sleep and mood in subjects with high screen-time use – a family-based pilot experiment and large-scale experimental study.

Version: Version 1

Date: 25.02.2019

Ethics project id: S-20170213

PI: Anders Grøntved

Table of contents

Preface 3
Introduction..3
Screen-time use among children and adults 3
High screen-time usage - a public health concern 4
Early childhood screentime – a unique public health concern 4
High screentime and sleep, mental health and physical activity 4
Investigating the effect of screen-time in a free-living setting 5
Methodology...5
Purpose of the pilot study 5
Purpose of the large-scale experiment 5
Hypotheses in the large-scale experiment 6
Definition of *screen-time* 6
Participants...7
Justification for including children aged 6-10 in the study 7
Inclusion criteria 9
Exclusion criteria from all measurements 9
Power calculation and randomization for the large-scale experiment 10

Recruitment of study participants in the pilot and the large-scale experiment 11
Insurance 12
Intervention 13
1st intervention: Complete removal of recreational screen-time 13
2nd intervention arm – pilot study, only: Decreased and timed recreational screen-time 13
3rd control arm – large intervention scale experiment, only: Practice recreational & work screen-time as usual 14
Tools to help the families comply with the intervention conditions 14
Evaluation of intervention 15
Process evaluation 15
Interview after completion of the intervention 15
Measurement methods 16
Sleep (only for participants ≥ 6 years of age) 16
Physiological stress 16
Physiological stress - Heart rate variability (only for participants ≥ 6 years of age) 16
Physiological stress - Awakening cortisol response and diurnal cortisol (adults) 17
Objectively measured physical activity 17
Smartphone and tablet/iPad usage 17
Television usage 18
Laptop and stationary computer usage 18
Short survey: Screen-based activity on devices we do not monitor objectively 19
Questionnaire: Mood (adults) 19
Questionnaire: Symptoms of depression (adults), pilot study only 19
Questionnaire: Subjective well-being (adults) 19
Questionnaire: Subjective wellbeing (children, large-scale experimental study 19
Questionnaire: Pain and discomfort (adults) 19
Questionnaire: Sleep Quality (adult, large-scale experimental study) 19
Biological material: Saliva samples 20
Financial incentive for participation in the intervention 20
Survey 20
Pilot and large-scale experiment 20
Publication of results 21

Ethical considerations of the project....21
Steps we will take to ensure that children are not subjected to any form of coercion....21
Consent and assent....21
Reasons for including minors in the study....22
Risks, side-effects and disadvantages associated with participation....22
Costs and benefits of study participation....22
Data safety....23
Financial support....23
Reference list....23

Preface

This ethical approval is for the large-scale experiment with the overall purpose to study the immediate short-term health risks of high use of screen media in children and adults. This large-scale experiment builds on the previously approved Pilot study by the Regional Scientific Ethical Committees for Southern Denmark (Project-ID: S-20170213 CSF). A request to the Regional Scientific Ethical Committees for Southern Denmark stated that it is sufficiently to send in an additional protocol describing the changes in the large-scale experiment including changes in the number of participants and purpose (mail received 22.11.2018, [redacted])

Introduction

Screen-time use among children and adults

Young people and adults spend a large and increasing amount of their daily time using various screen-based electronic media devices. In particular the use of new portable screen-based devices as smartphones and tablets and specific content usage such as social networking systems (e.g. Facebook) have had an extensive growth during the latest decade, and is dramatically changing the screen use volume and the way young people and adults entertain themselves and communicate and interact with friends, peers, family, and professional relations. Recently, the Digital Day 2016 study, a survey of the total media and communications activities of children and adults in the United Kingdom, was conducted. The results of the study showed that in children aged 6-11, average daily media and communications time was a staggering 4 hours and 49 minutes, during which 31 minutes included *media multitasking* (i.e. using multiple screen-based devices simultaneously, such as texting while watching television). Among children aged 11-15 and adults 16 years and above this was even higher, where average daily media and communications time was 6 hours and 20 minutes and 8 hours and 45 minutes, respectively, excluding media multitasking. There was a clear distinction in child and adult screentime behavior. Overall, total screen-based media use was accumulated while engaging in different types of activities; watching, listening, communicating, playing and reading or browsing. However, young children spent the majority of their screentime engaging in activities involving watching and playing, whereas adults to a larger extent spent their

screentime listening, communicating and browsing [1]. Clearly, today, both children and adults dedicate large amounts of time in everyday life engaging in activities on screen-based media devices. Additionally, children and adults exhibit distinct screentime behavior and should therefore receive our separate attention.

High screen-time usage - a public health concern

Early childhood screentime – a unique public health concern

Modern day's high use of screen-based media is discussed in the public in terms of its possible harmful effects on health and development of young people. Children's use of screen-based media is a clear emerging public health concern which requires serious attention and research to keep pace with the fast and profound changes in technology of screen media and how it is used is needed. Although there are many expected benefits of the use of today's screen-based media devices such as staying connected with friends and family, and increasing opportunities for learning different subjects and acquiring new skills [2], not much is known about the effects different patterns of screen time have on children's general health and wellbeing.

Recently, the Danish National Health Board published physical activity recommendations for children aged 0-4 years old [3]. One of the main recommendations to promote physical activity was to limit screen time. The reasoning behind recommending a limitation is the Danish National Health Boards review of the scientific literature, which concludes that sedentary behavior, mainly measured as TV-exposure, is associated with lower psychosocial development in children aged 0-4 years old [3]. The recommendation does not declare a specific amount of time that screen media use should be limited to because of a lack of evidence on this topic, in this particular age group [3]. Clearly, as children, are unique in the way they spend their time on screen-based media devices, high childhood screentime use presents a distinct public health concern. This concern is echoed among health professionals who are concerned about the long-term impact on growth and development high screentime may have for this young population group and thus not comparable with the potentially health consequences of high screentime in adults.

High screentime and sleep, mental health and physical activity

Recent experiments under controlled conditions in adults have investigated the effect of evening exposure to LED-backlit screens, which emit large doses of blue-wavelength light, on sleep quality and circadian physiology [4-6]. The studies showed a postponement of the evening rise in melatonin, an increased sense of alertness and poorer sleep quality, due to the LED-backlit screen exposure [4-6]. We are unaware of any experimental studies that have investigated the immediate health effects of limiting habitual screen-based media use among children and adults with high use, and this scarcity severely hinders our basic understanding of the effect of this behavior. It is not unlikely that the current habitual use of screen-based devices could affect circadian physiology and sleep patterns in adults, with medium-term consequences for mental health. Some experimental evidence suggests that computer gaming in young people increases sympathetic tone and mental workload [7], i.e. induces a somatic stress response. Today's screen-based media offers a wide range of opportunities for children and adults, including access to social media sites, gaming activities and much more. Engagement in these activities may acutely affect levels of enthusiasm, fear and other potent emotions, consequently leaving some individuals in an

almost perpetual state of emotional stress. Cross-sectional [8] and prospective [9] observational evidence suggests that some screen-based media use in young adulthood is associated with increased risk of depression. However, major methodological limitations exist in the observational research, which has been conducted to date. To the best out knowledge, no studies have investigated the effect of restricted screen-time on mental health and well-being. Rigorous experimental research is warranted to investigate the extent to which screen-based media affects mental health. Lastly, it is very likely that these screen-based media behaviors displace physical activity, which is known to have serious long-term implications for physical health of young people. Results from observational studies indicate a small negative association between screen-based media use and physical activity in young children and youth [10-12]. However, these studies have major methodological limitations and no experimental studies have investigated the immediate health effect of limiting habitual screen use. It is paramount that new, updated research is conducted to further understand the significance of heavy use of the screen-based media of the 21st century.

Investigating the effect of screen-time in a free-living setting

Previous studies that have investigated the biological effect of exposure to digital screens have been conducted in sleep laboratory settings [4-6]. These studies were conducted under heavily standardized conditions in terms of sleep (scheduled sleep), physical distance from screen-based device to the participants' face, posture, strength of external lighting and more. It would be valuable to investigate the physiological, mental and behavioral effect of a large restriction in screen-based media use under everyday psychological, social and environmental circumstances. Employing a rigorous experimental design, we may be able to answer important questions about causality; does habitual screen time affect sleep patterns, physical activity behavior and mental stress? The gain in knowledge from such a study will aid in the development of initiatives to lower screen-time among adults and children for whom screen-based media use is at a level detrimental to health. Furthermore, authorities and professionals within the field of public health will have strong experimentally backed-up scientific evidence to formulate guidelines on amount and timing of screen-based media for specific age groups.

Methodology

Purpose of the pilot study

The purpose of the pilot study part is to conduct a pilot experiment, which will serve as the foundation for the planning and execution of the large-scale experiment. The purpose of the pilot experiment is severalfold:

I. To assess the degree of compliance to the prescribed study conditions
II. To explore whether the measurements methods are feasible in a free-living setting
III. To evaluate effect sizes in order to assess the required sample size for the full-scale experiment
IV. To assess the time and cost of conducting the experiment

Purpose of the large-scale experiment

I. To investigate the immediate short-term effect of limiting use of screen-based media in the family during spare time on physical activity patterns, sleep time and sleep quality, and measures of wellbeing in children 4 to nearly 15 years of age where at least one parent have a high use of spare time screen media. (Children 4 to nearly 6 years of age will besides questionnaires only have their physical activity patterns measured and mental health based on a parent reported questionnaire)
II. To investigate the immediate short-term effect of limiting use of screen-based media on physical activity patterns, sleep time and sleep quality, and measures of stress and wellbeing among adults (parents) with a high use of spare time screen media.

Primary outcomes for the largescale experiment for children: Total activity, defined as "Any activity (including standing up) which cannot be characterized as sedentary time during waking hours, if we assume waking hours to be constant".

Primary outcomes for the largescale experiment for adults: "Total sleep duration" defined as time spent asleep within sleep period, excluding wake time. Secondarily if problems with measures, "Sleep period" indicates self-reported time from "fell asleep" to "woke up".

Secondary outcomes for the largescale experiment for children: Other measures of activity, sedentary time, sleep, physiological stress and wellbeing.

Secondary outcomes for the largescale experiment for adults: Measures of activity, sedentary time, other measures of sleep, musculoskeletal pain/discomfort, physiological stress and wellbeing.

Hypotheses in the large-scale experiment

Limiting use of screen-based media time will:

- Substitute time spent sedentary (sitting/lying down) with activity among the participating children and adults.
- Reduce latency to sleep onset and increase length of sleep and sleep stages among the participating children and adults.
- Decrease 24-hour HRV sympathetic tone among the adults as a measure of stress (taking time spent in physical activity increasing sympathetic tone into account).
- Increase salivary cortisol awaking response and lower bedtime cortisol levels in adults as a measure of stress.

Definition of *screen-time*

Screen-time is defined as being exposed in the direction of one's face to a digital screen emitting light. Screen-based media may be used for different purposes, such as for work or for recreational purposes. This includes watching television (from whichever distance one may choose to do so), using portable screen-based media devices (iPads, tablets, iPhones etc.) and more.

The following exposures are <u>not</u> considered screen-time in this project:

- Using an installed GPS in the car for directions
- Setting on or shutting off the alarm clock on one's portable screen-based media device.
- Having a conversation on a phone or other device that does not involve looking at a lit digital screen
- Listening to a voice mail message
- Short segments of screen-time from screens mounted in busses and other forms of public transportation, as well as other public venues. However, the consumption of screen-time in public places for extended periods of time is not permitted.

Participants

The goal is to recruit family households of 1-2 adults and 1-2 children, all of whom reside on the same address and registered on this address only. To be considered eligible at least one child must be between the age of six and ten. Based on our experience with the recruitment process in the pilot study for the experimental part of the study, interested eligible families for the experiment often have children younger or older than 6-10 years. Thus, we allow children 4-nearly 6 years of age to participate in physical activity measurements, sleep time (parent reported sleep time) and relevant questionnaires, whereas children up to nearly 15 years of age, are offered the same measurement package as children 6-10 years of age. We assume that it is important to offer these children to be participants so they can be a part of the family experiment equally to the other family members, and since including children 4 years of age will not be detrimental for parents measured sleep patterns and potentially other measures.

Justification for including children aged 6-10 in the study

Childhood - A time of growth and development

Compared to adults, young children are at a stage of life including growth and development. At this point, children develop both physically and mentally. Therefore, it is important to establish good conditions for children to mature optimally throughout their early years. This includes getting adequate sleep and being physically active. This is especially true because much concern surrounds the notion that behaviors relating to health in childhood are carried into young adulthood and adulthood. This concern is supported by findings from the scientific literature [13, 14]; it has e.g. been documented that physical activity tracks well across the lifespan, i.e. physical activity early in life influences physical activity later in life. This left researchers to underline the importance of increased physical activity in childhood [14]. Physical activity is an important feature of a healthy adult life, as physical activity is related to risk of type 2 diabetes [15] and cardiovascular disease [16]. Furthermore, children are in general thought to be more physically active than adults, which is supported by data from ´Idrættens Analyse Institut´ showing a higher proportion of children participating in sports and recreational physical activity during spare time compared to adults [17]. Also, the characteristics and the setting of the physical activity vary greatly according to age and developmental stage [18, 19]. Thus, compared to adults, who are busy with carrying out daily routines such as preparing meals and doing chores, we expect that removing high screen time in the spare time will predominantly increase physical activity in children, as a results of more potential time for free play.

As mentioned earlier much concern is expressed from health professional concerning children's high use of screen-based media devices. We would therefore like to include children in our study to investigate the effect a

heavy restriction in screen-based media use has on health parameters such as physical activity, time spent sedentary and sleep patterns for this particular age group. We cannot expect the same effects in adults and the age groups are at different stages of life biologically and mentally. Furthermore, because screen time most likely is an important cause of physical inactivity in children, it is crucial that the causal relationship between physical activity and screen time among children is investigated – as is our goal in this experiment.

Children and adults – different users of screen-based media

As written in the introduction, children and adults exhibit very different screen time behaviors in terms of content. Children spend their time playing games and watching video clips. Arguably, this type of screen-based media content is constructed to be particularly captivating of one's attention – especially for very young children, who by their very nature are more susceptible to external influence. It is therefore clear that these two age groups require our separate attention when it comes to understanding their screen time and the consequences may have for their health. Our experiment includes a heavy restriction in screen-based media use and the impact this may have on physical activity, sedentary behavior, sleep and more. Because children and adults have distinct patterns of screen time use, we cannot expect that the effect on the measurement outcomes in question from restricting this behavior will be the same for children and adults. Therefore, including both children and adults in our study is necessary to understand the difference in effect of the intervention on the outcome measurements.

How minors benefit especially from participation in this study

As mentioned earlier, children are generally more physically active than adults. However, much physical activity is displaced by screen time in children with high screen time. Therefore, if those children participate in our experimental study, they can potentially engage in a wealth of health-enhancing physical activity. Thus, there is more unlocked potential for children compared to adults with high screen time, when it comes to engaging in physical activity. Furthermore, there is much debate in the news media concerning the relationship between high screen time and sleep in young children. It may be the case that children's sleep is affected by evening screen-time, which may have implications for their long-term growth and development. Therefore, compared to mature adults, children may benefit more in terms of sleep quality and sleep duration from participation in our experimental study.

Furthermore, on a societal level, children more generally benefit from the gain in knowledge from our experimental study. National recommendations for physical activity for children 5-17 years of age thus far does not include a recommendation to decrease screen time. This shortcoming arguably is due to lack of evidence within this area. If young children participate in our study, we hopefully will add important knowledge to the research field of screen time, physical activity and health – knowledge which can be utilized as part of national and international recommendations for minors. Lastly, our findings could be used as a tool to construct future interventions which can help decrease high screen time for those children for whom their use is a problem.

Including younger and not older children

The following are arguments for including younger children in the study, which are related to the feasibility of the study:

- Young children compared to older children are thought to be more responsive to parents or caretakers, who, besides acting as powerful role models, are still in control of children's everyday life. Therefore, to test our hypothesis, it is necessary to include children for whom the intervention can fit into their every social structure of life.
- When the children get older the usage of screen based socializing networking systems to communicate and connect with friends increases dramatically and participating in this study the older child will miss out on social interaction on the social media with his/her friends likely having a larger personal consequence and resulting in a negative impact on the compliance to the study.

Inclusion criteria

- High screen time according to self-report:
  In each household, at least one adult must be above the 50th percentile for self-reported screen-time during spare time based on what was reported in the survey ((characterized as high in this study).
- To be eligible for the measurements children in the household must be $\geq$ 4 and < 15 years old during the entire experiment period.
- Adults must work full time or be full-time students
- Adults and children who participates in the measurements must have the resources to remove all recreational- and work-/school-related screen-time in the late afternoon and evening hours and during weekend days, with a few exceptions (described in the "intervention" section below), for a period of 2 weeks (intervention length).
- The children in the household must be $\geq$ 4 years of age.
- The household must include at least one adult and one child 6-10 years of age at the time the survey was sent out, both of whom must consent to participate in the experiment.
-
- Participants must report to be particularly motivated to decrease screen-time for the whole family household.
- All participating children and at least one participating parent must be capable to hand-over smartphones and tablets during the intervention period (two weeks).
- Members of the household who choose not to participate or who is ineligible to participate in the measurements or the intervention, must be willing to support the remainder of the household in making the experiment a success for them.

We will assess all of the above in a survey (Appendix A) combined with a personal phone call following the survey (described further in "Recruitment of study participants" below)

Exclusion criteria from all measurements

- If the adults or children only reside in the household part time, i.e. have multiple addresses

- Prescribed a period of sick leave due to stress within the last 3 months.
- Diagnosis of sleep disorders from their general practitioner still interfering sleep
- Working night shifts
- In any shape or form limited in one's ability to participate in habitual physical activity
- Neuropsychiatric disorders, such as Attention Deficit Hyperactivity Disorder (ADHD) and autism
- Already participating in other experimental studies (intervention studies)

We will assess all of the above in a survey (Appendix A) or during a personal phone call following the survey (described further in "Recruitment of study participants" below)

Participants pilot study

The goal is to recruit n=24 households for the pilotstudy, which may vary in terms of having one or two adults or children. The number of participants included is determined mainly by the primary purpose of the experiment; to test the feasibility of an experiment and its methods. When analyzing the effect sizes in the pilot experiment we will evaluate mostly the direction of the effect measures and the variation of these changes, rather than the width of the associated confidence intervals. The information from the pilot study, e.g. estimated effects sizes and the number of participants, will be used to make power calculations for the large-scale experiment. Therefore, it may happen that this study may be slightly underpowered, but this is on purpose as the main goal is not to evaluate effect sizes, but rather to evaluate feasibility of a study and its methods.
If too few households volunteer to participate in the study in the pilot study and the largescale experiment, we may additionally recruit from forsøgsperson.dk, a social media site (e.g. Facebook) or from schools via an application pinned on e.g. a news board for the students and their parents to see (Appendix B).

Power calculation and randomization for the large-scale experiment

Power calculation adults (primary outcome is total sleep time)
We define a 20 minutes/day increase in total sleep time as the approximate threshold for clinical relevance and used this in our power calculations. Based on a previous study among adults we expect a standard deviation of 40 minutes/day for total sleep time [20]. Because we randomize by family (not individual) we need to take into account a possible clustering of effect on sedentary time within children from each family. From our pilot study we expect that 1.6 adults will be enrolled per family. Assuming this average cluster size and an intraclass correlation coefficient of 0.25 for spousal total sleep time, we need to have a minimum of 45 families in each group to have a power of 80% to detect a difference in total sleep time of 20 minutes/day. To allow for the primary outcome data to be unavailable for 15 percent of participants we need to include a total number of 106 families with an expected 170 adults.

Power calculation children (primary outcome is sedentary time)
The primary outcome for children is not sedentary time but active time (see Purposes for the large-scale experiment), but since these measures are highly related we assume that the power calculations will be identical and based on sedentary time. From our pilot study we expect that 1.2 children will be enrolled per family, which provides us with an expected 127 children in total. With a total of 127 children from 106 families we have a power of 80% to detect a difference in daily sedentary time of 20 minutes/day assuming an intraclass correlation

coefficient of 0.40 for sibling sedentary time [21]. We consider an effect size lower than this as doubtfully clinically relevant.

Families will be randomized in random permuted blocks, potentially stratified by siblings, sex, age, or whether the whole or only part of the family participate in the experiment to either the intervention group reducing recreational screen time or the control group in a 1:1 ratio. Using the computer program ´OPEN randomise´ the random number sequence will be generated by an independent data manager from OPEN, securing allocation concealment. Blinding of the participants and the research staff is not possible after group allocation. However, most of the outcomes are objective and collected over more days and cannot be influenced by the observer. All adjudicators of outcomes will be blinded.

Recruitment of study participants in the pilot and the large-scale experiment

Please consult appendix C and C1 for an overview of the recruitment strategy and the meetings with the participants during the pilot study and largescale experiment, respectively.
For the pilot study a survey including the participant cover letters (Appendix A) will be sent out via e-boks to households in the municipality of Middelfart on the island of Fune, where one adult at minimum and at least one child between the age of 6 and 10 resides. Preliminary findings from the pilot study shows that 23,5% of the 1675 questionnaires in the survey was completed. Around 30% of the responders (112/394) showed interest in the experiment and of those 27 were eligible to be contacted to hear more about the experiment. Family recruitment and participating to the experiment in the pilot study is expected to be around ≈0.6% (10/1675*100%).

For the large-scale experiment, it has been estimated, that we will have to send out the survey including participant cover letters (Appendix A1) to all families with children 6-10 years of age, at the following Municipalities: Odense, Nordfyn, Faaborg-Midtfyn, Kerteminde, Nyborg, Svendborg, Vejle and Kolding (n≈35.000). This is based on our experience form the pilot study and a search on "Statistikbanken Denmark" counting the numbers of families with children 6-10 year of age. The exact number will be given by "Sundhedsdatastyrelsen". In the cover letter in Appendix A1, we inform participants that we plan to collect information on ethnicity (oprindelsesland) and socioeconomic/educational status from Danish registers. We plan to collect information from participants and non-participants to better describe our sample. If we succeed on this questions on ethnicity will be removed from the survey (Appendix A1).

The survey will be sent out in smaller surveys so participants signing up for the experiment, do not have to wait too long to get started, and so we only send out the survey including the questions concerning the invitation part to hear more about the experiment as long as recruitment for the experiment is necessary (Appendix A1). The survey will be sent out via e-boks with Region South Denmark or Southern University of Denmark as sender. Appendix C1 gives an overview of the recruitment process. Appendix A1a includes the standardized emails that will be send out to participants that we cannot reach by phone and interested participants not included in the experiment.

For the pilot study and the large-scale experiment only one adult and one child between 6-10 years of age will be included in the survey. If there are more than one of either, one of each will be selected at random. The survey will include questions about screen-time behavior, as well as correlates to screen-time and demographic factors.

The adult will answer on behalf of the child. Furthermore, in the survey, the participants are encouraged to install a recently developed application for IOS and Android devices (described in the "Smartphone and tablet/iPad usage" usage section in the "Methodology"), which quantifies the screen activity of these devices.

For the pilot study and the large-scale experiment there is a dual purpose of the survey; firstly, to collect data in order to in detail describe screen-time behavior for a specified population group from a larger geographical area; secondly, to serve as a platform for recruitment of study participants for the pilot and the large-scale experiment. We will ask specifically in the survey about whether or not the respondent, as well as the remaining members of the household, are interested in participating in an experimental study. The survey will include several questions that will allow the researchers to conduct a preliminary screening for eligibility.
The adults who will receive the survey may share their address with one other adult or live as the only adult in the household. To be eligible the adults must be in custody of and share the household with at least 1 child, who are between six and ten years of age, when the survey is sent out.

Participants who have reported that they are interested and are potentially eligible to participate in the experiment will be contacted by telephone to verify their interest in participating in the study and to assess whether the participants are fully eligible; e.g. we will assess whether or not the family members have chronic conditions (described earlier) that exclude them from the measurement part in the study. If more respondents are interested and eligible to participate in the experimental study than we plan to recruit, we will at random recruit a sample of the respondents. All interested participants will receive an email when recruitment for the experimental study is over thanking the families for their interest (see Appendix A1a). At the end of the phone call, the first meeting in the participants' home will be arranged. If there are two parents in the household, they must both be present at the meeting. If there is only one adult in the household, we will remind him or her that that he or she has the right to have another person present. At the meeting, the experiment and its methods will be explained more fully and in a pedagogical way (Appendix T1). Written participant information describing the experiment (Appendix D and Appendix D1) as well as consent forms (Appendix E) will be handed out. The researchers will also observe if the child/children in the household shows/show any sign of coercion from the parents to participate (read more about this in the "Steps we will take to ensure that children are not subjected to any form of coercion" section). At the meeting, a second meeting will be arranged approximately a week later. The time between the first and the second meeting will allow the participants to thoroughly contemplate whether or not they wish to participate in the experiment, after having met the researchers and read the written participant information describing the study. At the second meeting, the researchers will retrieve signed written informed consent forms from all members of the family who are interested to participate in the measurements and intervention. After this point, the recruitment process has finalized and the experiment itself begins.

Insurance

Injuries/ any harm that occurs in healthy subjects at research institutions, including the University of Southern Denmark are covered by two laws.
Workers' Compensation Act (Consolidated Act 2013-03-14 no. 278
Workers § 2, paragraph 1) Law on Complaints and Claims access in the health sector (Consolidated Act 2011-11-07 no. 1113 § 19, paragraph 2): Supplement to the Workers' Compensation Act, this covers any differential requirements (requirements that are larger than what is covered by Workers' Compensation Act).

Intervention

The current pilot study is a 2-week two-arm (arm 1 & arm 2) randomized study with no control group whereas the large-scale experiment is a 2-week two-arm (arm 1 & arm 3) randomized controlled study with one control group, conducted under free living conditions. The intervention arms include removing recreational screen-time (as well as any screen-time for entertainment purposes at work), although they differ in terms of the scale of removal.

The randomization in the pilot study will be performed 1:1, meaning that there is an equal chance of being allocated to either intervention arm. We may stratify the randomization by age and gender of the family members, to assure comparability between the two arms on every other parameter than the intervention.

1st intervention: Complete removal of recreational screen-time

Under this study condition participants must remove all recreational screen-time. This includes exclusion of any form of screen-time in the household, at friend's and family's house and so forth. As a tool to comply with the conditions of this intervention, the families will hand-over all portable screen-based media devices, such as smartphones, iPads, gamer computers etc. not used at work, school or to other vital functions, to the researchers. The portable devices will securely be stored in locked safes at the institute of Sports Science and Clinical Biomechanics. In return, each participant who before had a telephone will receive a phone which basically can only make phone calls and text message (Appendix U1). For a maximum of ½ an hour a day, participants may use this device for absolute necessary contact. This includes, but it not limited to:

- Contact with co-workers, friends and family that is necessary for a functional everyday life. Such contact could include sms texting to make arrangements, e.g. playdates for one's children or to arrange a dinner party with friends. Also, parents whose children are involved in sports may be involved in the planning sporting events. It also includes contact with one's partner about daily planning, including picking up children, grocery shopping etc.
- Checking whether or not a text message received necessarily requires an answer, i.e. relates to planning of everyday life. If not, a short message should be sent to the person who sent the text to remind them that he or she is part of the intervention.

Beyond this <½ hour/day of necessary recreational screen-time, the families are allowed 3 hours/week of recreational screen-based media use for entertainment purposes in the household.
To keep up with national and international news, participants may listen to a non-screen based radio transmitter. They may also listen to music from non-screen based devices, such as cd-players.

2nd intervention arm – pilot study, only: Decreased and timed recreational screen-time

Under this study condition, participants must remove all recreational screen-time before a certain hour in the evening. For the adults, screen-time is not allowed from 20 o'clock or 2 hours before bedtime, i.e. if one goes to bed at 21 o'clock no screen time is permitted from 19 o'clock. Habitual sleep time will be determined based on

sleep monitoring at baseline (see "sleep" section under "Measurement methods"). For the children, screen-time must be stopped from 18 o'clock or 2 hours before bedtime. There are no restrictions on the amount of screen-time before these designated hours. Thus, before these hours, the participants must continue their habitual screen-time completely. However, after the designated hours, before bedtime, screen-time must be removed completely.
If the participants want to listen to national and international news after the specified hours where screen-time is no longer permitted, a non-screen based radio transmitter may be used.

3rd control arm – large intervention scale experiment, only: Practice recreational & work screen-time as usual

To measure the effect of the intervention a control group is included. The participants in this group have no restrictions on the amount of screen-time, but are encouraged to keep their recreational and work screen time habits as usual. As with the intervention arms we will attempt to measure all activity on screen-based devices in the household in one way another. This surveillance is meant mainly as a tool for the researchers to measure compliance to the control group, but it may also help the participants themselves better adhere to the control conditions, knowing that their screen-time behavior is being monitored.

Tools to help the families comply with the intervention conditions

We will provide the study participants with several tools in attempt to maximize participant compliance to the interventions. Because the participants in the study report high use of screen-based media devices, it is important that they are provided with tools so they can manage and get accustomed to the experiment protocol. This is especially important as the intervention takes place in the household, with minor researcher presence. We will provide the following:

- **For both intervention arms:** Prior to the intervention we will hand out a sheet (Appendix F or F1) to the study participants concerning preparation for the intervention. The participants are encouraged to reflect and take notes on what type of activities to do to replace screen-based media devices. This will also include planning for times during the intervention that can be especially challenging, where screen-based media devices are normally used to soothe a stressful situation. Furthermore we will state that we strongly advise participants to explain to everyone in their social network, including friends and family, that they, for the next two weeks, are participating in an intervention study where the goal is to heavily reduce recreational screen time. Hence, they will not be on e.g. social media as much and only necessary digital contact will be possible. An option could be to schedule when to meet and be social during the 2 weeks.
- We will handout a small cardboard box with the letters "screen-based media hotel" written on it, in which the family can store portable screen-based media devices, during hours when screen-time is not permitted or when devices in intervention arm 1 are not used for work, school or other vital purposes. The box should be placed in close vicinity to, by not centrally in, the room where the family typically spends most of their time. This is because the family should be able to be reached if necessary, but they should not have their devices immediately in front of them, as this might introduce frequent temptation for use and the risk of "spontaneous" usage.

- We will attempt to measure all activity on screen-based devices in the household in one way another. This surveillance is meant mainly as a tool for the researchers to measure compliance to the intervention, but it may also help the participants themselves better adhere to the intervention conditions, knowing that their screen-time behavior is being monitored.
- We will hand out and help hang up standardized “signs” (Appendix G (G1) and H) e.g. in the household and at the workplace. In the household, these signs should be placed in rooms where the family spends most of their time. These “signs” will include reminders of the content and restrictions of the intervention. These should preferably be hanged up close to screen-based media devices. The goal of these “signs” is to serve as environmental cues for the participants, so they are frequently reminded of their participation in, as well as the content of, the intervention. This is among other things an attempt to remove “spontaneous” usage of screen-based devices, because one is simply accustomed to using these without much hesitation or thought.
- **For the 2nd intervention arm:** We will also create small “signs” that can be attached directly to one’s smartphone or tablet/iPad.
- **For the 2nd intervention arm:** We will ask participants not to engage in any group-messaging conversation, where they will receive messages that 1) are not necessary for everyday function and 2) may not even be for them specifically. Another suggestion will be to remove all unnecessary notifications on the phone, in attempt to remove all temptation during times when screen-time is not permitted.

Evaluation of intervention

Process evaluation

- Pilot study

Around a bi-weekly basis a person from the research team will call the families and ask them how the intervention is working out. We will ask questions based on a semi-structured interview guide. There is a dual purpose of these phone calls; first, to motivate and facilitate the participants to adhere to the intervention terms and secondly; to evaluate to what degree, how, why or why not the intervention has been adopted and implemented as intended. The conversations are planned to be relatively short and if suggested or requested by the participants, reduced to only one phone call the second week.

Interview after completion of the intervention

- Pilot study and the large-scale experimental study

Following completion of the baseline measurements and the intervention, a member of the research team will conduct an interview with members of the family, to assess the overall experience with the measurements and the intervention. The purpose of this is to understand the feasibility and acceptability of the measurements and the intervention. In the pilot study we will ask questions based on a semi-structured interview guide (Appendix P and Q).
In the large scale experimental study, we will ask the participating adults to answer a questionnaire (adult & children or adult only) after completion of the intervention (Appendix Q1).

The endpoint of the pilot part is to evaluate what aspects of the measurement period and intervention that works and what aspects require improvement in a full-scale experiment, which will include severalfold more participants. This interview is an important part of the formative evaluation of the pilot experiment.
This formative evaluation will be used to formalize a randomized trial to test the effects of a user-centered motivational intervention on reducing screen time in at large scale setting.

Measurement methods

Appendix I provide an overview of the content of the pilot experiment. Appendix C1 provides adjustments to the large-scale experimental study. Below is a specification of each of the measurement methods planned to be included in the study. Appendix R and R1 provides the parent and child logs, that the parents must fill out during baseline and follow up measurements for the pilot-, and the large scale experimental study, respectively. Finally, an updated version of the participant manual from the pilot study (Appendix J) has been included for the large-scale experimental study (Appendix J1).

The children and adults will not undergo the same measurement protocol. The adults will undergo a more extensive examination compared to the children, as different hypothesis will be addressed and practical matters must be taken into consideration. When only adults undergo a specific measurement or when the measurement will only be carried out in the large-scale experimental study, this has been stated in parenthesis below in each subheading. For example, only the adults will be involved in the collection of saliva samples

Sleep (only for participants ≥ 6 years of age)

We will utilize recently developed methodology to accurately assess sleep in free-living (home-based) conditions. This technology is based on single-channel electroencephalogram (EEG)-based system that is unobtrusive to wear during sleep with automatic algorithm-based scoring of sleep stages (wake, light sleep, deep sleep, rapid eye movement (REM) sleep) on a 30-second epoch basis [22, 23]. The monitoring includes mounting three electrodes to the back of the head, immediately before bedtime. Three wires will connect the electrodes to a small portable device, which stores the data throughout the night (Appendix J p. 4-7, Appendix J1 p. 5-9.
Baseline sleep data will be used to assess habitual sleep patterns, which is relevant for the planning of the 2nd intervention arm (pilot study).

Physiological stress (adults)

To study the effect of screen time restriction on the two main stress response pathways, the sympathetic adrenal medullary axis and the hypothalamic pituitary adrenal cortex axis, we will obtain continuous 24-hour measures of heart rate variability [24] and salivary samples of cortisol at morning rise and shortly hereafter [25].

Physiological stress - Heart rate variability (adults)

Heart rate variability will be measured by mounting two electrodes on the upper body (Appendix J p.12-14, Appendix J1 p.16-20) where an EKG measurement device will be attached. If the child experiences skin

irritation due to the patches where the device will be attached, they are allowed to carry out this measurement every second day instead of tree days in a row according to the protocol.

Physiological stress - Awakening cortisol response and diurnal cortisol (adults)

Known as the Cortisol Awakening Response, production of cortisol has a circadian rhythm with peak levels in the early morning. To measure the morning response of cortisol, saliva samples will be collected immediately at awakening and then again 30, and 45 minutes later [25] (Appendix J p. 7-11, Appendix J1 p. 10-16). The samples will be stored in the participants' freezer until collected by the researchers. In addition, the adult will deliver one saliva sample in the evening hours. Thus, a total of four saliva samples per day will be collected. We will also look at the cortison response.

Objectively measured physical activity

We will include objectively measured physical activity to assess the impact of manipulating screen-based use (or removal thereof) on daily physical activity patterns. The motion sensor (the Axivity AX3) combines a micro electromechanical systems accelerometer, light and temperature sensor. The Axivity® AX3 monitor is a small (23 x 32.5 x 7.6 mm), lightweight (11 g), waterproof, 3-axis accelerometer data logger (Axivity, 2016).

We will measure physical activity by attaching motion sensors (Axivity AX3 monitor) to the study participants with tape (Opsite Flexifix®) directly on the body. In-between the instrument and the skin a folded piece of soft bandage (Mesoft kompres usteril 5*5 cm) is placed. The motion sensor is taped to the skin in a way that allows fresh air to the area below and the monitor ("plaster metoden"). This method has so far been applied with success in the Danish family based study: Lolland-Falster Undersøgelsen (LOFUS).

Using the above described method to attach the monitor to the skin has shown to reduce potential skin irritation previously reported (se UNDERRETNING OM ALVORLIGE BIVIRKNINGER/HÆNDELSER I HENHOLD TIL KOMITÉLOVEN § 30, (IKKE-LÆGEMIDDELFORSØG, September the 20th).

The monitors will be attached on the participants right thigh and to the right on the lower back to enable full 24-hour recordings. Compared to previously used methods using this method will likely improve compliance rates, improve the control of non-wear time and the quality of measurement since the accelerometer keeps the same position on the body and follows the movement of the body, making it easier to estimate time spent in different positions such as sitting and lying important for the aim of this study.

If the participants have eczema or other skin problems, or the participants due to other yet unknown circumstances prefer to have the monitors attached to the skin with belts, we will give the participants that opportunity. "Bæltemetoden"; one belt worn around the stomach and one belt worn around the thigh (Appendix J p. 15-20, Appendix J1 p. 21-28). We expect that the proportion of participants using either method will be even distributed in the control and intervention arm.

Smartphone and tablet/iPad usage

To monitor smartphone, tablet and iPad usage, the study participants will install a recently developed application for IOS (Appendix K) and android (Appendix L) or Appendix J1 p. 34-36, developed by the software developing company Centic. The application measures when a screen is active and by doing so, we will be able to quantify

the use of portable screen-based media use on the units on which the application is installed. By using this newly developed application we will besides measuring baseline conditions to a large extent be able to measure the degree of compliance to the study conditions. Ideally, we would measure every portable screen-based device that the study participants would potentially use in their spare time. The application is developed for research purposes and data is only stored at the University of Southern Denmark servers.
The above most likely will only apply to those who are randomized to the 2nd intervention- or control arm, as the majority of all portable screen-based devices – i.e. the devices we are able to measure via the application preferable will be handed over to the researchers for participants who are randomized to the 1st intervention arm.

Television usage

In addition to smartphone, iPad and Tablet usage, we also want to be able to measure television-viewing, to more fully assess recreational screen-time. This will be done by attaching two small photosensors to every television screen in the household. These small devices detect whether or not the screen is emitting light and store information about this throughout the intervention. The researchers will mount this onto the televisions at the beginning of the study. The sensors will be equipped with a small detector, which shows if the photosensor has been removed. This measure is taken so that it is possible to detect if participants either deliberately or accidently remove the photosensors, so we will be able to detect loss of information and the underlying reason for this.

Laptop and stationary computer usage

To measure laptop and stationary computer usage in the household, we will choose one of the two following options (both of which applies to all laptop and stationary computers):

1) The participants will be instructed to install time management software on their laptop and stationary computers in the household. The software summarizes amount of use, hence giving an overview of the total use. The software does not send the data of the computer usage to a third party and is only stored locally. Immediately following the intervention the participants must send the data from the software to us researchers, to document their use (or lack thereof) during the two week intervention.
2) At our research unit, our engineer and sports scientist will develop software which is comparable to the app software to smartphones, tablets and iPads (described earlier), only that this software collects data from a computer.

It is the plan that the app, the TV monitors and the time management software will be installed or connected to the screen devices during the whole experiment period in all the intervention arms including the control group making it possible to quantify degree of compliance and detect any changes in the screen time during the different stages in the experiment period and to reduce participant burden.

Short survey: Screen-based activity on devices we do not monitor objectively

Every evening the parents must report in a short survey (Appendix M or M1) if any members of the household have used a screen-based device in the recreational hours that we are not able to measure objectively. For the first intervention arm, any use of such a device would actually be a breach of the rules of the intervention.
By making the study participants complete this short daily questionnaire our goal is to be able to quantify degree of total compliance to the intervention. We are e.g. unable to measure screen-time on devices at friends and families' houses. Thus, if the study participants report that they had screen-time on one or several of the above-mentioned devices, we will be able to quantify this from the logbook. Furthermore, participants receive a sheet where they can keep track on the amount of legal screen time (Appendix M1).

Questionnaire: Mood (adults)

Mood will be measured using the profile of mood scale (POMS) (Appendix N), which is a well-validated self-reported measure of mood states with high internal consistency [26]. Answers provide standardized scores for six identified subscales: Tension-Anxiety, Anger-Hostility, Fatigue-Inertia, Depression-Dejection, Vigor-Activity and Confusion-Bewilderment.

Questionnaire: Symptoms of depression (adults), pilot study only

Symptoms of depression will be assessed using the Major Depression Inventory (MDI) [27] (Appendix N), which has previously been used in our laboratory [9]. MDI consists of 12 items answered on a six-point Likert scale (0 to 5), which measure how often the symptoms have been present the last 14 days. MDI show a high content validity and internal consistency [27].

Questionnaire: Subjective well-being (adults)

We will measure subjective well-being using the WHO-5 inventory (Appendix N), which consists of five simple and non-intrusive questions. The questionnaire has been used in many studies in fields of inquiry, and has shown adequate validity [28].

Questionnaire: Subjective wellbeing (children, large-scale experimental study

Wellbeing will be measured using the parent reported Strengths and Difficulties Questionnaire (Appendix N1), which is a well-validated self-reported measure of wellbeing or mental health in children. We plan to use the subscales \`internalising´-, \`externalising´- and \`prosocial´ problems subscales and weelbeing & function (http://www.sdqinfo.com/a0.html). We include the original and follow up versions for children.

Questionnaire: Pain and discomfort (adults)

The participants will report experienced pain and discomfort during the last 14 days (Appendix N). This will include musculoskeletal pain, tiredness, headaches, sleep-related issues, and emotional state. The questions have been used in a large Danish population-based cross-sectional study – the Danish National Health Profile (Danish: Den Nationale Sundhedsprofil).

Questionnaire: Sleep Quality (adult, large-scale experimental study)

Leeds Sleep Evaluation Questionnaire. Two questions on participant reported sleep quality answered on a VAS scale is included. The answers are given on the basis of last night's sleep.

Biological material: Saliva samples

In each household a maximum of 45 saliva samples will be collected during the experiment, assuming 1.85 adults per family on average. The purpose of collecting the salivary samples is to analyze evening and morning cortisol and cortison, in relation to heavily restricted or timed screen-time. A salivary sample typically contains 0.5-1ml of material. Half of these will be collected immediately before the intervention (baseline) and the second half will be collected during the three final days of the intervention (follow-up). The adults in the household – not the researchers - will be in charge of collecting the salivary samples (Appendix J p. 7-11, Appendix J1 p. 10-16). Shortly after sampling the saliva, the sample will be stored in a fridge in the household. Following each of these procedures, a member of the research team will transport the frozen samples in a safe Medical Cold box from the household to the laboratories at the Institute of Sports Science and Clinical Biomechanics. Thus, we will create a research biobank here. The purpose of this biobank is to store the salivary samples for a very short period around a year before they are sent to a laboratory for analysis. The samples will be stored in the biobank until all measurements from all the participants in the experiment have been gathered. The amount of time the samples will be stored will depend on the timing of the baseline and follow-up measurements, for each family. The samples will be sent to the Department of Clinical Biochemistry at Slagelse Medical Hospital for analyses. Samples will be destroyed immediately after analysis.

Financial incentive for participation in the intervention

Survey

Those respondents who complete the survey and install the application will participate in a lottery to win 6000, 4000 or 2000 Danish kroner, which are tax free. It is well-established that response rates to populations-based studies often are low and selection biased. Therefore, we find it necessary to offer such financial incentives in attempt to increase response rates. By doing so, we will have more rich descriptive data available compared to what is currently available in the scientific literature. The amounts specified above for the pilot study are similar to those offered in a large Danish population-based cross-sectional study – the Danish National Health Profile (Danish: Den Nationale Sundhedsprofil). In the large-scale experiment, respondents, who complete the survey, will participate in a lottery to win 2500 and 1000 Danish kroner, which are tax free. If the participants also install the application on one smartphone, they will participate in a lottery to get another 2000 Danish kroner, which are also tax free. We plan to send out the survey 3 times including one or more Municipalities each time with the possibility to participate in a similar lottery. We will provide no other goods for the participants, financial or otherwise, than what is described above.

Pilot and large-scale experiment

Because the study requires some significant degree of resource allocation for the family to collect measurements as well as to comply with the intervention, we find it necessary to offer a financial incentive to those households who complete the intervention. We estimate that the participants may need to allocate up to 10 hours of their spare time to this project. Completion of the intervention refers to not dropping out, e.g. not choosing to halfway through the intervention. Therefore, those who complete the intervention are offered 1000 Danish kroner, which is taxable. Those who do not complete the intervention will not be offered 1000 Danish kroner, nor will they receive an amount of the 1000 Danish kroner proportional to the length of their participation. We will provide no other goods for the participants, financial or otherwise, than what is described above. In the large scale-

experiment this amount of money will be 500 Danish kroner, which is taxable. The children will receive a diploma for participating (Appendix A1).

Publication of results

Independent of the direction of the findings (positive or negative) or whether the findings are inconclusive, the results of the current study will be made public to the scientific community and to other interested readers. It will be made public via peer-reviewed medical or other health-related journals, as well as be presented at scientific conferences, nationally and internationally.

Ethical considerations of the project

Steps we will take to ensure that children are not subjected to any form of coercion

The verbal information for the children and their parents (or legal guardian) and the consent form will stipulate that participation in the project is voluntary, that their identity will be protected, and that they can withdraw (their participation and data) from the project whenever they wish. There will be no inducement, coercion or perceived pressure to participate. Parents are instructed to inform their child of the nature, purpose and the type of data collected in the study, so that they can give consent in advance to the extent that their capabilities allow. Young children's capacity for understanding theoretical information about the purpose, benefits, risks and consequences of participating in a study will be very limited. Thus, the consent of the youngest children needs to be sought continuously throughout the study whenever data is being collected. The test personnel in this project will be trained and instructed in detecting signs of child dissent. The training will be conducted by a researcher with extensive experience in doing research involving children of young ages, including having knowledge of important ethical considerations in this regard. Thus, the researcher in charge of the training will have substantial knowledge within ethical standards and child psychology, in relating to relevant research procedures. Indicators of child dissent includes: request to terminate/withdraw, increased latency of responses, turning to parent, avoidance of eye contact and withdrawal into self. If a child is showing signs of dissent all test personnel will be instructed not to proceed with any assessments.

Consent and assent

The parents will be thoroughly informed about the extent of physical measurements and surveillance included in the current project. They will be asked to provide consent on the behalf of their child (appendix E and E1 includes the consent forms). Any questions the participants may have at this stage, or any other stage, in relation to the monitoring procedure, will be answered promptly by researchers involved in the study.
We will in the pilot study as well as in the large-scale experiment collect a written, marked with a data and signed consent form from both (all) parents in custody of the child. If a parent in custody of the child cannot be present when signing the consent form on behalf of the child, the research staff will secure that a written and signed parent power of attorney form is provided by the absent parent (see appendix W1).

Reasons for including minors in the study

The current study includes young children who are known to spend much of their spare time on screen-based media devices. Children's use of screen-based devices is heavily discussed in the news media in terms of its potentially harmful effects. There is a strong concern in the public and among health professionals that heavy use of devices such as iPads and smartphones has a negative impact on children's psychological and physiological health. This is also related to the concern that heavy use is especially harmful in young children, as it might establish a behavior which is carried into young adulthood, and negatively impact psychological and physiological development in the process. However, very little basic research on this topic is available. It is therefore quintessential that studies are conducted which address this issue, to further our understanding of potential harmful effects. This knowledge is important as it will be evidence for the necessity of creating intervention which aims to effectively lower screen-time usage among this population group.
Because we are including minors in the study, we have deliberately chosen to include non-invasive measurement methods. Therefore, minors will not be subjected to any measurements which are considered intrusive and uncomfortable.

Risks, side-effects and disadvantages associated with participation

We consider no risk or particular disadvantages associated with participation in the study.

During the study, we will attempt to assess the participants' screen time via multiple sources. Importantly, our surveillance is very superficial in the sense that we are not collected detailed information about the participants' screen-time activity. We are only assessing whether or not a device is or has been active, not the content and type of usage on the specific device. The only minor exception is that with those individuals who own an Android device, we will be able to quantify the proportional use of different types of Aps, hence to some extent mapping the type of screen-time usage. However, we will not be able state for what purpose an app has been used. We will explain this to the participants and re-iterate it if necessary.
The participants – both children and adults - will be wearing an EKG device for three consecutive days before the intervention and during the three remaining days of the intervention. At the same time, the participants will be wearing two motion sensors, as well as EEG equipment on the back of the head during nighttime. Therefore, there is some burden of wearing a significant amount of equipment all at once, although it is only for three days. If the participants show any signs of discomfort with wearing the equipment, we will remind them that their participation is voluntary and that they may remove the equipment – which is easily removable – at any time. Some of our own internal work with the sleep monitoring equipment suggests that very few participants find discomfort when wearing the equipment.

We do not consider it relevant to discuss side-effects in the current study.

Costs and benefits of study participation

Participation in the study will require allocation of time resources for the family for approximately three weeks (baseline measurements prior to the intervention, the intervention itself, and the follow-up measurements). During baseline and follow-up, which are identical in terms of measurement protocol content and length (3

days), there is some participant burden, as described extensively earlier (please see appendix J, and appendix J1 for an overview of measurement methods). However, the participants go through the experiment in the comfort of their own home and are thus not required to transport themselves to the researchers. Also, the methods should not be considered invasive, as e.g. blood sampling would be. In terms of benefits, the vast gain in knowledge from this experiment arguably up weighs any potential burden, although this burden should be considered minor.

Data safety

Data collected in the current study will be reported to the Danish Data protection agency through SDU rio, according to Danish law. The management and analysis of data in the current study will comply fully with the Danish Personal Data law.

Financial support

Associate professor and research leader Anders Grøntved conceived the idea for the project and developed the original grant application. The current study is financed by a grant from the European Research Council (ERC) Advanced Grants, being 1.5 million Euro (See budget in appendix O). Please consult the budget for a description of for how the grant finances the current study. Anders Grøntved has no affiliation with the ECR other than the financial support. Anders Grøntved has had access to the grant from the 1st of February 2017. The grant has been placed on the account number [redacted] located at University of Southern Denmark, Department of Sports Science and Clinical Biomechanics. Thus, the grant will be at the departments disposal to finance the current projects activities.

Reference list

1. Ofcom, *Digital Day 2016 - Results from the children's diary study*. 2016.
2. O'Keeffe, G.S. and K. Clarke-Pearson, *The impact of social media on children, adolescents, and families.* Pediatrics, 2011. **127**(4): p. 800-4.
3. Sundhedsstyrelsen, *Sundhedsmæssige effekter af fysisk aktivitet og stillesiddende tid hos 0-4-årige børn: en systematisk litteraturgennemgang*, Sundhedsstyrelsen, Editor. 2016.
4. Bues, M., et al., *LED-backlit computer screens influence our biological clock and keep us more awake.* Journal of the Society for Information Display, 2012. **20**(5): p. 266-272.
5. Cajochen, C., et al., *Evening exposure to a light-emitting diodes (LED)-backlit computer screen affects circadian physiology and cognitive performance.* J Appl Physiol (1985), 2011. **110**(5): p. 1432-8.
6. Chang, A.M., et al., *Evening use of light-emitting eReaders negatively affects sleep, circadian timing, and next-morning alertness.* Proc Natl Acad Sci U S A, 2015. **112**(4): p. 1232-7.
7. Chaput, J.P., et al., *Video game playing increases food intake in adolescents: a randomized crossover study.* Am J Clin Nutr, 2011. **93**(6): p. 1196-203.
8. Casiano, H., et al., *Media Use and Health Outcomes in Adolescents: Findings from a Nationally Representative Survey.* Journal of the Canadian Academy of Child and Adolescent Psychiatry, 2012. **21**(4): p. 296-301.
9. Grontved, A., et al., *A prospective study of screen time in adolescence and depression symptoms in young adulthood.* Prev Med, 2015. **81**: p. 108-13.
10. Ekelund, U., et al., *TV viewing and physical activity are independently associated with metabolic risk in children: the European Youth Heart Study.* PLoS Med, 2006. **3**(12): p. e488.

11. Marshall, S.J., et al., *Relationships between media use, body fatness and physical activity in children and youth: a meta-analysis.* Int J Obes Relat Metab Disord, 2004. **28**(10): p. 1238-46.
12. Taveras, E.M., et al., *Longitudinal relationship between television viewing and leisure-time physical activity during adolescence.* Pediatrics, 2007. **119**(2): p. e314-9.
13. Fraser, B.J., et al., *Childhood Muscular Fitness Phenotypes and Adult Metabolic Syndrome.* Med Sci Sports Exerc, 2016. **48**(9): p. 1715-22.
14. Telama, R., *Tracking of physical activity from childhood to adulthood: a review.* Obes Facts, 2009. **2**(3): p. 187-95.
15. Smith, A.D., et al., *Physical activity and incident type 2 diabetes mellitus: a systematic review and dose-response meta-analysis of prospective cohort studies.* Diabetologia, 2016. **59**(12): p. 2527-2545.
16. Li, J. and J. Siegrist, *Physical activity and risk of cardiovascular disease--a meta-analysis of prospective cohort studies.* Int J Environ Res Public Health, 2012. **9**(2): p. 391-407.
17. Idrættens Analyseinstitut, *DANSKERNES MOTIONS- OG SPORTSVANER 2016*. 2016.
18. Pellegrini, A.D. and P.K. Smith, *Physical activity play: the nature and function of a neglected aspect of playing.* Child Dev, 1998. **69**(3): p. 577-98.
19. Strong, W.B., et al., *Evidence based physical activity for school-age youth.* J Pediatr, 2005. **146**(6): p. 732-7.
20. Al Khatib, H.K., et al., *Sleep extension is a feasible lifestyle intervention in free-living adults who are habitually short sleepers: a potential strategy for decreasing intake of free sugars? A randomized controlled pilot study.* Am J Clin Nutr, 2018. **107**(1): p. 43-53.
21. Pereira, S., et al., *Resemblance in physical activity levels: The Portuguese sibling study on growth, fitness, lifestyle, and health.* Am J Hum Biol, 2018. **30**(1).
22. Kaplan, R.F., et al., *Performance evaluation of an automated single-channel sleep-wake detection algorithm.* Nat Sci Sleep, 2014. **6**: p. 113-22.
23. Wang, Y., et al., *Evaluation of an automated single-channel sleep staging algorithm.* Nature and Science of Sleep, 2015. **7**: p. 101-111.
24. *Heart rate variability: standards of measurement, physiological interpretation and clinical use. Task Force of the European Society of Cardiology and the North American Society of Pacing and Electrophysiology.* Circulation, 1996. **93**(5): p. 1043-65.
25. Stalder, T., et al., *Assessment of the cortisol awakening response: Expert consensus guidelines.* Psychoneuroendocrinology, 2016. **63**: p. 414-32.
26. McNair, D.M., et al., *Profile of mood states*. 1971, San Diego, Calif.: Educational and Industrial Testing Service.
27. Bech, P., et al., *The sensitivity and specificity of the Major Depression Inventory, using the Present State Examination as the index of diagnostic validity.* J Affect Disord, 2001. **66**(2-3): p. 159-64.
28. Topp, C.W., et al., *The WHO-5 Well-Being Index: a systematic review of the literature.* Psychother Psychosom, 2015. **84**(3): p. 167-76.

Final study protocol

Title: Short-term efficacy of reducing screen media use on physical activity, sleep, and physiological stress in families with children aged 4–14 years

Version: Final version

Date: 10.12.2019

Ethics project id: S-20170213

Clinicaltrials.gov: NCT04098913

PI: Anders Grøntved

Table of contents

Introduction 3
Screen-time use among children and adults 3
High screen-time usage - a public health concern 3
Early childhood screen time – a unique public health concern 3
High screen time, sleep, mental health and physical activity 4
Investigating the effect of screen-time in a free-living setting 5
Methodology 5
Purpose of the study 5
Primary outcome 5
Secondary outcomes 5
Other exploratory outcomes 6
Hypotheses 6
Definition of *screen-time* 6
Participants 6
Justification for including children aged 6-10 in the study 7
Inclusion criteria 8

Exclusion criteria from all measurements 9
Power calculation and randomization 9
Randomization and blinding 10
Recruitment of study participants 10
Insurance 11
Intervention 12
Intervention arm: Limiting recreational screen-time to a minimum 12
Control arm: Practice recreational & work screen-time as usual 12
Tools to help the families comply with the intervention conditions 12
Evaluation of intervention 13
Interview after completion of the intervention 13
Measurement methods 13
Sleep (only for participants ≥ 6 years of age) 14
Physiological stress (adults) 14
Physiological stress - Heart rate variability (adults) 14
Physiological stress - Awakening cortisol response and diurnal cortisol (adults) 14
Objectively measured physical activity 14
Smartphone and tablet/iPad usage 14
Television usage 15
Laptop and stationary computer usage 15
Short survey: Screen-based activity on devices we do not monitor objectively 15
Questionnaire: Mood (adults) 16
Questionnaire: Subjective well-being (adults) 16
Questionnaire: Subjective wellbeing (children) 16
Questionnaire: Pain and discomfort (adults) 16
Questionnaire: Sleep Quality (adult) 16
Biological material: Saliva samples 16
Financial incentive for participation in the intervention 17
Survey 17
Randomized trial 17
Publication of results 17
Ethical considerations of the project 17
Steps we will take to ensure that children are not subjected to any form of coercion 17

Consent and assent 18
Reasons for including minors in the study 18
Risks, side-effects and disadvantages associated with participation.................................. 19
Costs and benefits of study participation 19
Data safety.. 19
Financial support.. 19
Reference list... 20
Study protocol revision sequence and summary of changes.. 22

Introduction

Screen-time use among children and adults

Young people and adults spend a large and increasing amount of their daily time using various screen-based electronic media devices. In particular the use of new portable screen-based devices as smartphones and tablets and specific content usage such as social networking systems (e.g. Facebook) have had an extensive growth during the latest decade, and is dramatically changing the screen use volume and the way young people and adults entertain themselves and communicate and interact with friends, peers, family, and professional relations. Recently, the Digital Day 2016 study, a survey of the total media and communications activities of children and adults in the United Kingdom, was conducted. The results of the study showed that in children aged 6-11, average daily media and communications time was a staggering 4 hours and 49 minutes, during which 31 minutes included *media multitasking* (i.e. using multiple screen-based devices simultaneously, such as texting while watching television). Among children aged 11-15 and adults 16 years and above this was even higher, where average daily media and communications time was 6 hours and 20 minutes and 8 hours and 45 minutes, respectively, excluding media multitasking. There was a clear distinction in child and adult screen time behavior. Overall, total screen-based media use was accumulated while engaging in different types of activities; watching, listening, communicating, playing and reading or browsing. However, young children spent the majority of their screen time engaging in activities involving watching and playing, whereas adults to a larger extent spent their screen time listening, communicating and browsing [1]. Clearly, today, both children and adults dedicate large amounts of time in everyday life engaging in activities on screen-based media devices. Additionally, children and adults exhibit distinct screen time behavior and should therefore receive our separate attention.

High screen-time usage - a public health concern

Early childhood screen time – a unique public health concern

Modern day's high use of screen-based media is discussed in the public in terms of its possible harmful effects on health and development of young people. Children's use of screen-based media is a clear

emerging public health concern which requires serious attention and research to keep pace with the fast and profound changes in technology of screen media and how it is used is needed. Although there are many expected benefits of the use of today's screen-based media devices such as staying connected with friends and family, and increasing opportunities for learning different subjects and acquiring new skills [2], not much is known about the effects different patterns of screen time have on children's general health and wellbeing. Recently, the Danish National Health Board published physical activity recommendations for children aged 0-4 years old [3]. One of the main recommendations to promote physical activity was to limit screen time. The reasoning behind recommending a limitation is the Danish National Health Boards review of the scientific literature, which concludes that sedentary behavior, mainly measured as TV-exposure, is associated with lower psychosocial development in children aged 0-4 years old [3]. The recommendation does not declare a specific amount of time that screen media use should be limited to because of a lack of evidence on this topic, in this particular age group [3]. Clearly, as children, are unique in the way they spend their time on screen-based media devices, high childhood screen time use presents a distinct public health concern. This concern is echoed among health professionals who are concerned about the long-term impact on growth and development high screen time may have for this young population group and thus not comparable with the potentially health consequences of high screen time in adults.

High screen time, sleep, mental health and physical activity

Recent experiments under controlled conditions in adults have investigated the effect of evening exposure to LED-backlit screens, which emit large doses of blue-wavelength light, on sleep quality and circadian physiology [4-6]. The studies showed a postponement of the evening rise in melatonin, an increased sense of alertness and poorer sleep quality, due to the LED-backlit screen exposure [4-6]. We are unaware of any experimental studies that have investigated the immediate health effects of limiting habitual screen-based media use among children and adults with high use, and this scarcity severely hinders our basic understanding of the effect of this behavior. It is not unlikely that the current habitual use of screen-based devices could affect circadian physiology and sleep patterns in adults, with medium-term consequences for mental health. Some experimental evidence suggests that computer gaming in young people increases sympathetic tone and mental workload [7], i.e. induces a somatic stress response. Today's screen-based media offers a wide range of opportunities for children and adults, including access to social media sites, gaming activities and much more. Engagement in these activities may acutely affect levels of enthusiasm, fear and other potent emotions, consequently leaving some individuals in an almost perpetual state of emotional stress. Cross-sectional [8] and prospective [9] observational evidence suggests that some screen-based media use in young adulthood is associated with increased risk of depression. However, major methodological limitations exist in the observational research, which has been conducted to date. To the best out knowledge, no studies have investigated the effect of restricted screen-time on mental health and well-being. Rigorous experimental research is warranted to investigate the extent to which screen-based media affects mental health. Lastly, it is very likely that these screen-based media behaviors displace physical activity, which is known to have serious long-term implications for physical health of young people. Results from observational studies indicate a small negative association between screen-based media use and physical activity in young children and youth [10-12]. However, these studies have major methodological limitations and no experimental studies have investigated the immediate health effect of limiting habitual screen use. It is

paramount that new, updated research is conducted to further understand the significance of heavy use of the screen-based media of the 21st century.

Investigating the effect of screen-time in a free-living setting

Previous studies that have investigated the biological effect of exposure to digital screens have been conducted in sleep laboratory settings [4-6]. These studies were conducted under heavily standardized conditions in terms of sleep (scheduled sleep), physical distance from screen-based device to the participants' face, posture, strength of external lighting and more. It would be valuable to investigate the physiological, mental and behavioral effect of a large restriction in screen-based media use under everyday psychological, social and environmental circumstances. Employing a rigorous experimental design, we may be able to answer important questions about causality; does habitual screen time affect sleep patterns, physical activity behavior and mental stress? The gain in knowledge from such a study will aid in the development of initiatives to lower screen-time among adults and children for whom screen-based media use is at a level detrimental to health. Furthermore, authorities and professionals within the field of public health will have strong experimentally backed-up scientific evidence to formulate guidelines on amount and timing of screen-based media for specific age groups.

Methodology

Purpose of the study

I. To investigate the short-term effect of limiting use of screen-based media in the family during leisure time on physical activity patterns, sleep time and sleep quality, and measures of wellbeing in children 4-14 years of age where at least one parent have a high use of spare time screen media.
II. To investigate the short-term effect of limiting use of screen-based media during leisure time on physical activity patterns, sleep time and sleep quality, and measures of stress and wellbeing among adults (parents) with a high use of spare time screen media.

Primary outcome

I. Between group change in children's leisure time spent being non-sedentary (min/day). Non-sedentary time is defined as any waking activity characterized as not being in a sitting, reclining or lying posture with minimal stationary movement.

Secondary outcomes

I. Between group change in leisure time spent being non-sedentary (adults).
II. Between group change in leisure time moderate to vigorous physical activity (MVPA) (children and adults).
III. Between group change in leisure time spent being non-sedentary on weekdays (children and adults).
IV. Between group change in leisure time spent being non-sedentary on weekend days (children and adults).

V. Between group change in total sleep duration (children and adults).
VI. Between group change in sleep onset latency (children and adults).
VII. Between group change in wake after sleep onset (children and adults).
VIII. Between group change in sleep stages (awake, light sleep, deep sleep, REM-sleep) (children and adults).

Other exploratory outcomes

I. Between group change in cortisol awakening response (adults).
I. Between group change in diurnal cortisol slope (adults).
II. Between group change in heart rate variability adults).
III. Between group change in non-metabolic heart rate variability (adults).
IV. Between group change in mood disturbance score (adults).
V. Between group change in WHO-5-score (adults).
VI. Between group change in the total difficulties score (children).
VII. Between group change in subjective sleep quality (adults).

Hypotheses

Restricting leisure screen media use to an amount much below habitual levels for a period of two weeks increases leisure time spent being non-sedentary, increases total sleep duration and decreases markers of physiological stress, in families of adults and children.

Definition of *screen-time*

Screen-time is defined as being exposed in the direction of one's face to a digital screen emitting light. Screen-based media may be used for different purposes, such as for work or for recreational purposes. This includes watching television (from whichever distance one may choose to do so), using portable screen-based media devices (iPads, tablets, iPhones etc.) and more.

The following exposures are not considered screen-time in this project:

- Using an installed GPS in the car for directions
- Setting on or shutting off the alarm clock on one's portable screen-based media device.
- Having a conversation on a phone or other device that does not involve looking at a lit digital screen
- Listening to a voice mail message
- Short segments of screen-time from screens mounted in busses and other forms of public transportation, as well as other public venues. However, the consumption of screen-time in public places for extended periods of time is not permitted.

Participants

The goal is to recruit family households of 1-2 adults and 1-2 children, all of whom reside on the same address and registered on this address only. To be considered eligible at least one child must be between the age of six and ten. Based on our experience with the recruitment process in the pilot study

for the experimental part of the study, interested eligible families for the experiment often have children younger or older than 6-10 years. Thus, we allow children 4-nearly 6 years of age to participate in physical activity measurements, sleep time (parent reported sleep time) and relevant questionnaires, whereas children up to nearly 15 years of age, are offered the same measurement package as children 6-10 years of age. We assume that it is important to offer these children to be participants so they can be a part of the family experiment equally to the other family members, and since including children 4 years of age will not be detrimental for parents measured sleep patterns and potentially other measures.

Justification for including children aged 6-10 in the study

Childhood - A time of growth and development

Compared to adults, young children are at a stage of life including growth and development. At this point, children develop both physically and mentally. Therefore, it is important to establish good conditions for children to mature optimally throughout their early years. This includes getting adequate sleep and being physically active. This is especially true because much concern surrounds the notion that behaviors relating to health in childhood are carried into young adulthood and adulthood. This concern is supported by findings from the scientific literature [13, 14]; it has e.g. been documented that physical activity tracks well across the lifespan, i.e. physical activity early in life influences physical activity later in life. This left researchers to underline the importance of increased physical activity in childhood [14]. Physical activity is an important feature of a healthy adult life, as physical activity is related to risk of type 2 diabetes [15] and cardiovascular disease [16]. Furthermore, children are in general thought to be more physically active than adults, which is supported by data from ´Idrættens Analyse Institut´ showing a higher proportion of children participating in sports and recreational physical activity during spare time compared to adults [17]. Also, the characteristics and the setting of the physical activity vary greatly according to age and developmental stage [18, 19]. Thus, compared to adults, who are busy with carrying out daily routines such as preparing meals and doing chores, we expect that removing high screen time in the spare time will predominantly increase physical activity in children, as a results of more potential time for free play. As mentioned earlier much concern is expressed from health professional concerning children's high use of screen-based media devices. We would therefore like to include children in our study to investigate the effect a heavy restriction in screen-based media use has on health parameters such as physical activity, time spent sedentary and sleep patterns for this particular age group. We cannot expect the same effects in adults and the age groups are at different stages of life biologically and mentally. Furthermore, because screen time most likely is an important cause of physical inactivity in children, it is crucial that the causal relationship between physical activity and screen time among children is investigated – as is our goal in this experiment.

Children and adults – different users of screen-based media

As written in the introduction, children and adults exhibit very different screen time behaviors in terms of content. Children spend their time playing games and watching video clips. Arguably, this type of screen-based media content is constructed to be particularly captivating of one's attention – especially for very young children, who by their very nature are more susceptible to external influence. It is therefore clear that these two age groups require our separate attention when it comes to understanding

their screen time and the consequences may have for their health. Our experiment includes a heavy restriction in screen-based media use and the impact this may have on physical activity, sedentary behavior, sleep and more. Because children and adults have distinct patterns of screen time use, we cannot expect that the effect on the measurement outcomes in question from restricting this behavior will be the same for children and adults. Therefore, including both children and adults in our study is necessary to understand the difference in effect of the intervention on the outcome measurements.

How minors benefit especially from participation in this study

As mentioned earlier, children are generally more physically active than adults. However, much physical activity is displaced by screen time in children with high screen time. Therefore, if those children participate in our experimental study, they can potentially engage in a wealth of health-enhancing physical activity. Thus, there is more unlocked potential for children compared to adults with high screen time, when it comes to engaging in physical activity. Furthermore, there is much debate in the news media concerning the relationship between high screen time and sleep in young children. It may be the case that children's sleep is affected by evening screen-time, which may have implications for their long-term growth and development. Therefore, compared to mature adults, children may benefit more in terms of sleep quality and sleep duration from participation in our experimental study. Furthermore, on a societal level, children more generally benefit from the gain in knowledge from our experimental study. National recommendations for physical activity for children 5-17 years of age thus far does not include a recommendation to decrease screen time. This shortcoming arguably is due to lack of evidence within this area. If young children participate in our study, we hopefully will add important knowledge to the research field of screen time, physical activity and health – knowledge which can be utilized as part of national and international recommendations for minors. Lastly, our findings could be used as a tool to construct future interventions which can help decrease high screen time for those children for whom their use is a problem.

Including younger and not older children

The following are arguments for including younger children in the study, which are related to the feasibility of the study:

- Young children compared to older children are thought to be more responsive to parents or caretakers, who, besides acting as powerful role models, are still in control of children's everyday life. Therefore, to test our hypothesis, it is necessary to include children for whom the intervention can fit into their every social structure of life.
- When the children get older the usage of screen based socializing networking systems to communicate and connect with friends increases dramatically and participating in this study the older child will miss out on social interaction on the social media with his/her friends likely having a larger personal consequence and resulting in a negative impact on the compliance to the study.

Inclusion criteria

- High screen time according to self-report:

In each household, at least one adult must be above the 40th percentile for self-reported screen-time during spare time based on what was reported in the survey ((characterized as high in this study).

- To be eligible for the measurements children in the household must be ≥ 4 and < 15 years old during the entire experiment period.
- Adults must work full time or be full-time students
- Adults and children who participates in the measurements must have the resources to remove all recreational- and work-/school-related screen-time in the late afternoon and evening hours and during weekend days, with a few exceptions (described in the "intervention" section below), for a period of 2 weeks (intervention length).
- The children in the household must be ≥ 4 years of age.
- The household must include at least one adult and one child 6-10 years of age at the time the survey was sent out, both of whom must consent to participate in the experiment.
- Participants must report to be particularly motivated to decrease screen-time for the whole family household.
- All participating children and at least one participating parent must be capable to hand-over smartphones and tablets during the intervention period (two weeks).
- Members of the household who choose not to participate or who is ineligible to participate in the measurements or the intervention, must be willing to support the remainder of the household in making the experiment a success for them.

We will assess all of the above in a survey (Appendix A) combined with a personal phone call following the survey (described further in "Recruitment of study participants" below)

Exclusion criteria from all measurements

- If the adults or children only reside in the household part time, i.e. have multiple addresses
- Prescribed a period of sick leave due to stress within the last 3 months.
- Diagnosis of sleep disorders from their general practitioner still interfering sleep
- Working night shifts
- In any shape or form limited in one's ability to participate in habitual physical activity
- Neuropsychiatric disorders, such as Attention Deficit Hyperactivity Disorder (ADHD) and autism
- Already participating in other experimental studies (intervention studies)

We will assess all of the above in a survey or during a personal phone call following the survey (described further in "Recruitment of study participants" below)

Power calculation and randomization

Power calculation children (primary outcome is non-sedentary time)
We expect to include 1.96 children per family. Based on data from our pilot, we expect a standard deviation of 57 min/week for average change in non-sedentary time from baseline to follow-up in the experimental group and 39.7 min/week for the control group. Also, based on other internal work in children 0–17 years of age, we expect a 0.3 correlation between sibling non-sedentary time. In regard to

clinical relevancy, a cross-over study found that intermittent interruptions of walking, amounting to a total of 18 min during 3 hours of sitting, resulted in favorable metabolic changes, when compared to sitting only, in children 10.2 (1.5) years of age [20]. For the current study, a 24-min change is deemed a clinically relevant effect size, based on the expected between-group difference and project resources. Therefore, assuming an intraclass correlation coefficient of 0.3 for sibling non-sedentary time and a cluster size of 1.96 children per family, to detect a 24 min/day difference with a power of 80% and $\alpha = 0.05$, a total of 88 families including 174 children is required for the analyses.

Randomization and blinding

Families will be randomized in random permuted blocks (block size 2-4) to either the intervention group reducing recreational screen time or the control group in a 1:1 ratio. Using the computer program ´OPEN randomise´ the random number sequence will be generated by an independent data manager from OPEN, securing allocation concealment. Blinding of the participants and the research staff is not possible after group allocation. However, most of the outcomes are objective and collected over more days and cannot be influenced by the observer. All adjudicators of outcomes will be blinded.

Recruitment of study participants

Findings from the pilot study shows that 23,5% of the 1675 questionnaires in the survey was completed. Around 30% of the responders (112/394) showed interest in the experiment and of those 27 were eligible to be contacted to hear more about the experiment. Family recruitment and participating to the experiment in the pilot study is expected to be around ≈0.6% (10/1675*100%). For the study, we estimated, that we will have to send out the survey including participant cover letters to all families with children 6-10 years of age, at the following Municipalities: Odense, Nordfyn, Faaborg-Midtfyn, Kerteminde, Nyborg, Svendborg, Vejle and Kolding (n≈35.000). This is based on our experience form the pilot study and a search on "Statistikbanken Denmark" counting the numbers of families with children 6-10 year of age. The exact number will be given by "Sundhedsdatastyrelsen". In the cover letter, we inform participants that we plan to collect information on ethnicity (oprindelsesland) and socioeconomic/educational status from Danish registers. We plan to collect information from participants and non-participants to better describe our sample. If we succeed on this questions on ethnicity will be removed from the survey.

The survey will be sent out in smaller surveys so participants signing up for the experiment, do not have to wait too long to get started, and so we only send out the survey including the questions concerning the invitation part to hear more about the experiment as long as recruitment for the experiment is necessary. The survey will be sent out via e-boks with Region South Denmark or Southern University of Denmark as sender.

Only one adult and one child between 6-10 years of age will be included in the survey. If there are more than one of either, one of each will be selected at random. The survey will include questions about screen-time behavior, as well as correlates to screen-time and demographic factors. The adult will answer on behalf of the child. Furthermore, in the survey, the participants are encouraged to install a recently developed application for IOS and Android devices (described in the "Smartphone and

tablet/iPad usage" usage section in the "Methodology"), which quantifies the screen activity of these devices.

There is a dual purpose of the survey; firstly, to collect data in order to in detail describe screen-time behavior for a specified population group from a larger geographical area; secondly, to serve as a platform for recruitment of study participants for the study. We will ask specifically in the survey about whether or not the respondent, as well as the remaining members of the household, are interested in participating in an experimental study. The survey will include several questions that will allow the researchers to conduct a preliminary screening for eligibility.
The adults who will receive the survey may share their address with one other adult or live as the only adult in the household. To be eligible the adults must be in custody of and share the household with at least 1 child, who are between six and ten years of age, when the survey is sent out.

Participants who have reported that they are interested and are potentially eligible to participate in the experiment will be contacted by telephone to verify their interest in participating in the study and to assess whether the participants are fully eligible; e.g. we will assess whether or not the family members have chronic conditions (described earlier) that exclude them from the measurement part in the study. If more respondents are interested and eligible to participate in the experimental study than we plan to recruit, we will at random recruit a sample of the respondents. All interested participants will receive an email when recruitment for the experimental study is over thanking the families for their interest. At the end of the phone call, the first meeting in the participants' home will be arranged. If there are two parents in the household, they must both be present at the meeting. If there is only one adult in the household, we will remind him or her that that he or she has the right to have another person present. At the meeting, the experiment and its methods will be explained more fully and in a pedagogical way. Written participant information describing the experiment as well as consent forms will be handed out. The researchers will also observe if the child/children in the household shows/show any sign of coercion from the parents to participate (read more about this in the "Steps we will take to ensure that children are not subjected to any form of coercion" section). At the meeting, a second meeting will be arranged approximately a week later. The time between the first and the second meeting will allow the participants to thoroughly contemplate whether or not they wish to participate in the experiment, after having met the researchers and read the written participant information describing the study. At the second meeting, the researchers will retrieve signed written informed consent forms from all members of the family who are interested to participate in the measurements and intervention. After this point, the recruitment process has finalized and the experiment itself begins.

Insurance

Injuries/ any harm that occurs in healthy subjects at research institutions, including the University of Southern Denmark are covered by two laws. Workers' Compensation Act (Consolidated Act 2013-03-14 no. 278 Workers § 2, paragraph 1) Law on Complaints and Claims access in the health sector (Consolidated Act 2011-11-07 no. 1113 § 19, paragraph 2): Supplement to the Workers' Compensation Act, this covers any differential requirements (requirements that are larger than what is covered by Workers' Compensation Act).

Intervention

The intervention takes place in the families' household during everyday activities. The intervention requires that the family make several changes during everyday life, regarding screen time habits, during leisure, for 2 weeks.

Intervention arm: Limiting recreational screen-time to a minimum

Under this study condition participants must remove most recreational screen-time. This includes exclusion of any form of screen-time in the household, at friend's and family's house and so forth. As a tool to comply with the conditions of this intervention, the families will hand-over all portable screen-based media devices, such as smartphones, iPads, gamer computers etc. not used at work, school or to other vital functions, to the researchers. The portable devices will securely be stored in locked safes at the Department of Sports Science and Clinical Biomechanics. In return, each participant who before had a telephone will receive a phone which basically can only make phone calls and text message. For a maximum of ½ an hour a day, participants may use this device for absolute necessary contact. This includes, but it is not limited to:

- Contact with co-workers, friends and family that is necessary for a functional everyday life. Such contact could include sms texting to make arrangements, e.g. playdates for one's children or to arrange a dinner party with friends. Also, parents whose children are involved in sports may be involved in the planning sporting events. It also includes contact with one's partner about daily planning, including picking up children, grocery shopping etc.
- Checking whether or not a text message received necessarily requires an answer, i.e. relates to planning of everyday life. If not, a short message should be sent to the person who sent the text to remind them that he or she is part of the intervention.

Beyond this <½ hour/day of necessary recreational screen-time, the families are allowed 3 hours/week of recreational screen-based media use for entertainment purposes in the household.
To keep up with national and international news, participants may listen to a non-screen based radio transmitter. They may also listen to music from non-screen based devices, such as cd-players.

Control arm: Practice recreational & work screen-time as usual

To investigate the effect of the intervention a control group is included. The participants in this group have no restrictions on the amount of screen-time, but are encouraged to keep their recreational and work screen time habits as usual. As with the intervention arms we will attempt to measure all activity on screen-based devices in the household in one way another. This surveillance is meant mainly as a tool for the researchers to measure compliance to the control group, but it may also help the participants themselves better adhere to the control conditions, knowing that their screen-time behavior is being monitored.

Tools to help the families comply with the intervention conditions

We will provide the study participants with several tools in attempt to maximize participant compliance to the interventions. Because the participants in the study report high use of screen-based media

devices, it is important that they are provided with tools so they can manage and get accustomed to the experiment protocol. This is especially important as the intervention takes place in the household, with minor researcher presence. We will provide the following:

- Prior to the intervention we will hand out a sheet to the study participants concerning preparation for the intervention. The participants are encouraged to reflect and take notes on what type of activities to do to replace screen-based media devices. This will also include planning for times during the intervention that can be especially challenging, where screen-based media devices are normally used to soothe a stressful situation. Furthermore, we will state that we strongly advise participants to explain to everyone in their social network, including friends and family, that they, for the next two weeks, are participating in an intervention study where the goal is to heavily reduce recreational screen time. Hence, they will not be on e.g. social media as much and only necessary digital contact will be possible. An option could be to schedule when to meet and be social during the 2 weeks.
- We will attempt to measure all activity on screen-based devices in the household in one way another. This surveillance is meant mainly as a tool for the researchers to measure compliance to the intervention, but it may also help the participants themselves better adhere to the intervention conditions, knowing that their screen-time behavior is being monitored.
- We will hand out and help hang up standardized "signs" e.g. in the household and at the workplace. In the household, these signs should be placed in rooms where the family spends most of their time. These "signs" will include reminders of the content and restrictions of the intervention. These should preferably be hanged up close to screen-based media devices. The goal of these "signs" is to serve as environmental cues for the participants, so they are frequently reminded of their participation in, as well as the content of the intervention. This is among other things an attempt to remove "spontaneous" usage of screen-based devices, because one is simply accustomed to using these without much hesitation or thought.

Evaluation of intervention

Interview after completion of the intervention

Following completion of the baseline measurements and the intervention, a member of the research team will conduct an interview with members of the family, to assess the overall experience with the measurements and the intervention. We will ask the participating adults to answer a questionnaire (adult & children or adult only) after completion of the intervention. This formative evaluation will be used to formalize a randomized trial to test the effects of a user-centered motivational intervention on reducing screen time in at large scale setting.

Measurement methods

The children and adults will not undergo the same measurement protocol. The adults will undergo a more extensive examination compared to the children, as different hypothesis will be addressed and practical matters must be taken into consideration. When only adults undergo a specific measurement, this has been stated in parenthesis below in each subheading. For example, only the adults will be involved in the collection of saliva samples

Sleep (only for participants ≥ 6 years of age)

We will utilize recently developed methodology to accurately assess sleep in free-living (home-based) conditions. This technology is based on single-channel electroencephalogram (EEG)-based system that is unobtrusive to wear during sleep with automatic algorithm-based scoring of sleep stages (wake, light sleep, deep sleep, rapid eye movement (REM) sleep) on a 30-second epoch basis [21, 22]. The monitoring includes mounting three electrodes to the back of the head, immediately before bedtime. Three wires will connect the electrodes to a small portable device, which stores the data throughout the night.

Physiological stress (adults)

To study the effect of screen time restriction on the two main stress response pathways, the sympathetic adrenal medullary axis and the hypothalamic pituitary adrenal cortex axis, we will obtain continuous 24-hour measures of heart rate variability [23] and salivary samples of cortisol at morning rise and shortly hereafter [24].

Physiological stress - Heart rate variability (adults)

Heart rate variability will be measured by mounting two electrodes on the upper body where an EKG measurement device will be attached.

Physiological stress - Awakening cortisol response and diurnal cortisol (adults)

Known as the Cortisol Awakening Response, production of cortisol has a circadian rhythm with peak levels in the early morning. To measure the morning response of cortisol, saliva samples will be collected immediately at awakening and then again 30, and 45 minutes later [24]. The samples will be stored in the participants' freezer until collected by the researchers. In addition, the adult will deliver one saliva sample in the evening hours. Thus, a total of four saliva samples per day will be collected. We will also look at the cortison response.

Objectively measured physical activity

We will include objectively measured physical activity to assess the impact of manipulating screen-based use (or removal thereof) on daily physical activity patterns. The motion sensor (the Axivity AX3) combines a micro electromechanical systems accelerometer, light and temperature sensor. The Axivity® AX3 monitor is a small (23 x 32.5 x 7.6 mm), lightweight (11 g), waterproof, 3-axis accelerometer data logger (Axivity, 2016). We will measure physical activity by attaching motion sensors (Axivity AX3 monitor) to the study participants with elastic belts to enable full 24-hour recordings. One around the waist, where the sensor is placed such that, it is on the right hip facing away from the right of the body and a second belt is worn around the right thigh midway between the hip and the knee, where an accelerometer is placed in a pocket on this belt facing away from the body.

Smartphone and tablet/iPad usage

To monitor smartphone, tablet and iPad usage, the study participants will install a recently developed application for IOS and android, developed by the software developing company Centic. The application measures when a screen is active and by doing so, we will be able to quantify the use of

portable screen-based media use on the units on which the application is installed. By using this newly developed application we will besides measuring baseline conditions to a large extent be able to measure the degree of compliance to the study conditions. Ideally, we would measure every portable screen-based device that the study participants would potentially use in their spare time. The application is developed for research purposes and data is only stored at the University of Southern Denmark servers.

Television usage

In addition to smartphone, iPad and Tablet usage, we also want to be able to measure television-viewing, to more fully assess recreational screen-time. This will be done by using a small electronic device that has been developed by engineers at our department (Department of Sports Science and Clinical Biomechanics). Detecting TV usage is assessed by measuring the power cord current using a hall sensor. A hall sensor is a current sensor generating a voltage proportional to the current flowing through the sensor. The voltage is converted using an analog to digital converter with the installed micro-controller, in 1-min epocs. TV usage is detected by using a simple threshold, which is set substantial higher than the stand-by current (equal to or above the signal strength half-way between the minimum and maximal). By using this threshold, we also consider the fact that some TVs exhibit short burst of electrical activity, often during nocturnal hours. These signals, which are multiples above standby signal strength, but also multiples below television activity (which is often a 5-fold signal stronger than stand-by mode). The signals are characteristic of TV, which download information for the TV-users, when the TV is shut off. By using the defined cut-off above, TV-time is easily distinguished from these shorts burst of electrical activity not associated with TV usage. The researchers will mount this onto the televisions at the beginning of the study.

Laptop and stationary computer usage

To measure laptop and stationary computer usage in the household, software to measure computer use for OS X and Windows operating systems was developed comparable to the app software to smartphones and tablets and iPads (described earlier).

It is the plan that the app, the TV monitors and the PC software will be installed or connected to the screen devices during the whole experiment period in both study arms making it possible to quantify degree of compliance and detect any changes in the screen time during the different stages in the experiment period and to reduce participant burden.

Short survey: Screen-based activity on devices we do not monitor objectively

Every evening the parents must report in a short survey if any members of the household have used a screen-based device in the recreational hours that we are not able to measure objectively. For the first intervention arm, any use of such a device would actually be a breach of the rules of the intervention. By making the study participants complete this short daily questionnaire our goal is to be able to quantify degree of total compliance to the intervention. We are e.g. unable to measure screen-time on devices at friends and families' houses. Thus, if the study participants report that they had screen-time

on one or several of the above-mentioned devices, we will be able to quantify this from the logbook. Furthermore, participants receive a sheet where they can keep track on the amount of legal screen time.

Questionnaire: Mood (adults)

Mood will be measured using the profile of mood scale (POMS), which is a well-validated self-reported measure of mood states with high internal consistency [25]. Answers provide standardized scores for six identified subscales: Tension-Anxiety, Anger-Hostility, Fatigue-Inertia, Depression-Dejection, Vigor-Activity and Confusion-Bewilderment.

Questionnaire: Subjective well-being (adults)

We will measure subjective well-being using the WHO-5 inventory, which consists of five simple and non-intrusive questions. The questionnaire has been used in many studies in fields of inquiry, and has shown adequate validity [26].

Questionnaire: Subjective wellbeing (children)

Wellbeing will be measured using the parent reported Strengths and Difficulties Questionnaire, which is a well-validated self-reported measure of wellbeing or mental health in children. We plan to use the subscales `internalising´-, `externalising´- and `prosocial´ problems subscales and weelbeing & function (http://www.sdqinfo.com/a0.html). We include the original and follow up versions for children.

Questionnaire: Pain and discomfort (adults)

The participants will report experienced pain and discomfort during the last 14 days (Appendix N). This will include musculoskeletal pain, tiredness, headaches, sleep-related issues, and emotional state. The questions have been used in a large Danish population-based cross-sectional study – the Danish National Health Profile (Danish: Den Nationale Sundhedsprofil).

Questionnaire: Sleep Quality (adult)

Leeds Sleep Evaluation Questionnaire. Two questions on participant reported sleep quality answered on a VAS scale is included. The answers are given on the basis of last night's sleep.

Biological material: Saliva samples

In each household a maximum of 45 saliva samples will be collected during the experiment, assuming 1.85 adults per family on average. The purpose of collecting the salivary samples is to analyze evening and morning cortisol and cortison, in relation to heavily restricted or timed screen-time. A salivary sample typically contains 0.5-1ml of material. Half of these will be collected immediately before the intervention (baseline) and the second half will be collected during the three final days of the intervention (follow-up). The adults in the household – not the researchers - will be in charge of collecting the salivary samples. Shortly after sampling the saliva, the sample will be stored in a fridge in the household. Following each of these procedures, a member of the research team will transport the frozen samples in a safe Medical Cold box from the household to the laboratories at the Institute of Sports Science and Clinical Biomechanics. Thus, we will create a research biobank here. The purpose of this biobank is to store the salivary samples for a very short period around a year before they are sent to a laboratory for analysis. The samples will be stored in the biobank until all measurements from all

the participants in the experiment have been gathered. The amount of time the samples will be stored will depend on the timing of the baseline and follow-up measurements, for each family. The samples will be sent to the Department of Clinical Biochemistry at Slagelse Medical Hospital for analyses. Samples will be destroyed immediately after analysis.

Financial incentive for participation in the intervention

Survey

Those respondents who complete the survey and install the application will participate in a lottery to win 6000, 4000 or 2000 Danish kroner, which are tax free. It is well-established that response rates to populations-based studies often are low and selection biased. Therefore, we find it necessary to offer such financial incentives in attempt to increase response rates. By doing so, we will have more rich descriptive data available compared to what is currently available in the scientific literature. The amounts specified above for the pilot study are similar to those offered in a large Danish population-based cross-sectional study – the Danish National Health Profile (Danish: Den Nationale Sundhedsprofil). In the study, respondents, who complete the survey, will participate in a lottery to win 2500 and 1000 Danish kroner, which are tax free. If the participants also install the application on one smartphone, they will participate in a lottery to get another 2000 Danish kroner, which are also tax free. We plan to send out the survey 3 times including one or more Municipalities each time with the possibility to participate in a similar lottery. We will provide no other goods for the participants, financial or otherwise, than what is described above.

Randomized trial

Because the study requires some significant degree of resource allocation for the family to collect measurements as well as to comply with the intervention, we find it necessary to offer a financial incentive to those households who complete the intervention. We estimate that the participants may need to allocate up to 10 hours of their spare time to this project. Completion of the intervention refers to not dropping out, e.g. not choosing to halfway through the intervention. Therefore, the families who complete the study will receive a 500 DKK reimbursement for the time they put into completing the study (independent of group allocation). The children will receive a diploma for participating.

Publication of results

Independent of the direction of the findings (positive or negative) or whether the findings are inconclusive, the results of the current study will be made public to the scientific community and to other interested readers. It will be made public via peer-reviewed medical or other health-related journals, as well as be presented at scientific conferences, nationally and internationally.

Ethical considerations of the project

Steps we will take to ensure that children are not subjected to any form of coercion

The verbal information for the children and their parents (or legal guardian) and the consent form will stipulate that participation in the project is voluntary, that their identity will be protected, and that they

can withdraw (their participation and data) from the project whenever they wish. There will be no inducement, coercion or perceived pressure to participate. Parents are instructed to inform their child of the nature, purpose and the type of data collected in the study, so that they can give consent in advance to the extent that their capabilities allow. Young children's capacity for understanding theoretical information about the purpose, benefits, risks and consequences of participating in a study will be very limited. Thus, the consent of the youngest children needs to be sought continuously throughout the study whenever data is being collected. The test personnel in this project will be trained and instructed in detecting signs of child dissent. The training will be conducted by a researcher with extensive experience in doing research involving children of young ages, including having knowledge of important ethical considerations in this regard. Thus, the researcher in charge of the training will have substantial knowledge within ethical standards and child psychology, in relating to relevant research procedures. Indicators of child dissent includes: request to terminate/withdraw, increased latency of responses, turning to parent, avoidance of eye contact and withdrawal into self. If a child is showing signs of dissent all test personnel will be instructed not to proceed with any assessments.

Consent and assent

The parents will be thoroughly informed about the extent of physical measurements and surveillance included in the current project. They will be asked to provide consent on the behalf of their child. Any questions the participants may have at this stage, or any other stage, in relation to the monitoring procedure, will be answered promptly by researchers involved in the study.
We will collect a written, marked with a data and signed consent form from both (all) parents in custody of the child. If a parent in custody of the child cannot be present when signing the consent form on behalf of the child, the research staff will secure that a written and signed parent power of attorney form is provided by the absent parent.

Reasons for including minors in the study

The current study includes young children who are known to spend much of their spare time on screen-based media devices. Children's use of screen-based devices is heavily discussed in the news media in terms of its potentially harmful effects. There is a strong concern in the public and among health professionals that heavy use of devices such as iPads and smartphones has a negative impact on children's psychological and physiological health. This is also related to the concern that heavy use is especially harmful in young children, as it might establish a behavior which is carried into young adulthood, and negatively impact psychological and physiological development in the process. However, very little basic research on this topic is available. It is therefore quintessential that studies are conducted which address this issue, to further our understanding of potential harmful effects. This knowledge is important as it will be evidence for the necessity of creating intervention which aims to effectively lower screen-time usage among this population group.
Because we are including minors in the study, we have deliberately chosen to include non-invasive measurement methods. Therefore, minors will not be subjected to any measurements which are considered intrusive and uncomfortable.

Risks, side-effects and disadvantages associated with participation

We consider no risk or particular disadvantages associated with participation in the study.

During the study, we will attempt to assess the participants' screen time via multiple sources. Importantly, our surveillance is very superficial in the sense that we are not collected detailed information about the participants' screen-time activity. We are only assessing whether or not a device is or has been active, not the content and type of usage on the specific device.
The participants – both children and adults - will be wearing an EKG device for three consecutive days before the intervention and during the three remaining days of the intervention. At the same time, the participants will be wearing two motion sensors, as well as EEG equipment on the back of the head during nighttime. Therefore, there is some burden of wearing a significant amount of equipment all at once, although it is only for three days. If the participants show any signs of discomfort with wearing the equipment, we will remind them that their participation is voluntary and that they may remove the equipment – which is easily removable – at any time. Some of our own internal work with the sleep monitoring equipment suggests that very few participants find discomfort when wearing the equipment.

We do not consider it relevant to discuss side-effects in the current study.

Costs and benefits of study participation

Participation in the study will require allocation of time resources for the family for approximately three weeks (baseline measurements prior to the intervention, the intervention itself, and the follow-up measurements). During baseline and follow-up, which are identical in terms of measurement protocol content and length (3 days), there is some participant burden, as described extensively earlier. However, the participants go through the experiment in the comfort of their own home and are thus not required to transport themselves to the researchers. Also, the methods should not be considered invasive, as e.g. blood sampling would be. In terms of benefits, the vast gain in knowledge from this experiment arguably up weighs any potential burden, although this burden should be considered minor.

Data safety

Data collected in the current study will be reported to the Danish Data protection agency through SDU rio, according to Danish law. The management and analysis of data in the current study will comply fully with the Danish Personal Data law.

Financial support

Professor and research leader Anders Grøntved conceived the idea for the project and developed the original grant application. The current study is financed by a grant from the European Research Council (ERC) Advanced Grants, being 1.5 million Euro (See budget in appendix O). Please consult the budget for a description of for how the grant finances the current study. Anders Grøntved has no affiliation with the ECR other than the financial support. Anders Grøntved has had access to the grant from the 1st of February 2017. The grant has been placed on the account number [redacted] located at University of Southern Denmark, Department of Sports Science and Clinical Biomechanics. Thus, the grant will be at the departments disposal to finance the current projects activities.

Reference list

1. Ofcom, *Digital Day 2016 - Results from the children's diary study*. 2016.
2. O'Keeffe, G.S. and K. Clarke-Pearson, *The impact of social media on children, adolescents, and families.* Pediatrics, 2011. **127**(4): p. 800-4.
3. Sundhedsstyrelsen, *Sundhedsmæssige effekter af fysisk aktivitet og stillesiddende tid hos 0-4-årige børn: en systematisk litteraturgennemgang*, Sundhedsstyrelsen, Editor. 2016.
4. Bues, M., et al., *LED-backlit computer screens influence our biological clock and keep us more awake.* Journal of the Society for Information Display, 2012. **20**(5): p. 266-272.
5. Cajochen, C., et al., *Evening exposure to a light-emitting diodes (LED)-backlit computer screen affects circadian physiology and cognitive performance.* J Appl Physiol (1985), 2011. **110**(5): p. 1432-8.
6. Chang, A.M., et al., *Evening use of light-emitting eReaders negatively affects sleep, circadian timing, and next-morning alertness.* Proc Natl Acad Sci U S A, 2015. **112**(4): p. 1232-7.
7. Chaput, J.P., et al., *Video game playing increases food intake in adolescents: a randomized crossover study.* Am J Clin Nutr, 2011. **93**(6): p. 1196-203.
8. Casiano, H., et al., *Media Use and Health Outcomes in Adolescents: Findings from a Nationally Representative Survey.* Journal of the Canadian Academy of Child and Adolescent Psychiatry, 2012. **21**(4): p. 296-301.
9. Grontved, A., et al., *A prospective study of screen time in adolescence and depression symptoms in young adulthood.* Prev Med, 2015. **81**: p. 108-13.
10. Ekelund, U., et al., *TV viewing and physical activity are independently associated with metabolic risk in children: the European Youth Heart Study.* PLoS Med, 2006. **3**(12): p. e488.
11. Marshall, S.J., et al., *Relationships between media use, body fatness and physical activity in children and youth: a meta-analysis.* Int J Obes Relat Metab Disord, 2004. **28**(10): p. 1238-46.
12. Taveras, E.M., et al., *Longitudinal relationship between television viewing and leisure-time physical activity during adolescence.* Pediatrics, 2007. **119**(2): p. e314-9.
13. Fraser, B.J., et al., *Childhood Muscular Fitness Phenotypes and Adult Metabolic Syndrome.* Med Sci Sports Exerc, 2016. **48**(9): p. 1715-22.
14. Telama, R., *Tracking of physical activity from childhood to adulthood: a review.* Obes Facts, 2009. **2**(3): p. 187-95.
15. Smith, A.D., et al., *Physical activity and incident type 2 diabetes mellitus: a systematic review and dose-response meta-analysis of prospective cohort studies.* Diabetologia, 2016. **59**(12): p. 2527-2545.
16. Li, J. and J. Siegrist, *Physical activity and risk of cardiovascular disease--a meta-analysis of prospective cohort studies.* Int J Environ Res Public Health, 2012. **9**(2): p. 391-407.
17. Idrættens Analyseinstitut, *DANSKERNES MOTIONS- OG SPORTSVANER 2016*. 2016.
18. Pellegrini, A.D. and P.K. Smith, *Physical activity play: the nature and function of a neglected aspect of playing.* Child Dev, 1998. **69**(3): p. 577-98.
19. Strong, W.B., et al., *Evidence based physical activity for school-age youth.* J Pediatr, 2005. **146**(6): p. 732-7.
20. Belcher, B.R., et al., *Effects of Interrupting Children's Sedentary Behaviors With Activity on Metabolic Function: A Randomized Trial.* J Clin Endocrinol Metab, 2015. **100**(10): p. 3735-43.
21. Kaplan, R.F., et al., *Performance evaluation of an automated single-channel sleep-wake detection algorithm.* Nat Sci Sleep, 2014. **6**: p. 113-22.

22. Wang, Y., et al., *Evaluation of an automated single-channel sleep staging algorithm.* Nature and Science of Sleep, 2015. **7**: p. 101-111.
23. *Heart rate variability: standards of measurement, physiological interpretation and clinical use. Task Force of the European Society of Cardiology and the North American Society of Pacing and Electrophysiology.* Circulation, 1996. **93**(5): p. 1043-65.
24. Stalder, T., et al., *Assessment of the cortisol awakening response: Expert consensus guidelines.* Psychoneuroendocrinology, 2016. **63**: p. 414-32.
25. McNair, D.M., et al., *Profile of mood states*. 1971, San Diego, Calif.: Educational and Industrial Testing Service.
26. Topp, C.W., et al., *The WHO-5 Well-Being Index: a systematic review of the literature.* Psychother Psychosom, 2015. **84**(3): p. 167-76.

Study protocol revision sequence and summary of changes

25.02.2019	**Original ethics approved protocol version 1**
06.06.2019	**First family enrolled**
10.12.2019	**Final study protocol version**

Attachment of Axivity AX3 accelerometers were changed from using skin-tape to elastic belts due to experiences with skin irritation from other research projects in the research group.

Methodology for assessment of TV use was changed due to additional in-house methodological testing and developments

Symptoms of depression questionnaire (adults) was dropped based on experiences in the pilot study.

One inclusion criterion was slightly changed. Instead of at least one adult must be above the 50th percentile for self-reported screen-time during leisure time based on what was reported in the survey, this was changed to the 40th percentile as the cut off. This was done to safeguard enough families to be included, and we deemed this minor change of little relevance based on the first responses of the screen media use survey sent out to the population-based sample.

The final power calculation for the study was updated based on data from the pilot study.

All above amendments were done before first family was enrolled. Study details were registered in clinicaltrials.gov (NCT04098913) on September 23rd 2019 after four families (4.5% of families) had been enrolled in the study. One last additional amendment was implemented on December 12th 2019 by updating the list of outcomes in more detail to match the ones registered at clinical trials.gov. In this amendment we also changed the trial to include only one primary outcome (i.e. children's leisure time spent being non-sedentary (min/day)) instead of two. We added the second original primary outcome (i.e. adults total sleep duration) to the list of secondary outcomes. This was done because children's non-sedentary activity was deemed the most important outcome of interest.

30.03.2021	**Enrollment completed**
