# Supplementary material for: Screen Media Use and Mental Health of Children and Adolescents: A Secondary Analysis of a Randomized Clinical Trial
Source: JAMA Netw Open. 2024 Jul 12;7(7):e2419881. doi: 10.1001/jamanetworkopen.2024.19881 (PMC11245724; doi:10.1001/jamanetworkopen.2024.19881)
Supplement: Supplement 2. — eFigure. Flow Chart eTable 1. Comparison of Total Difficulties Scores at Baseline eTable 2. Analyses for Behavioral Strengths and Difficulties eTable 3. Comparison of Norm Data Proportions to Simulated Proportions eAppendix. [file jamanetwopen-e2419881-s002.pdf]

Supplementary Online Content

Schmidt-Persson J, Rasmussen MGB, Sørensen SO, et al. Screen media use and mental health of children and adolescents: a secondary analysis of a randomized clinical trial. *JAMA Netw Open.* 2024;7(7):e2419881. doi:10.1001/jamanetworkopen.2024.19881

eFigure. Flow Chart
eTable 1. Comparison of Total Difficulties Scores at Baseline
eTable 2. Analyses for Behavioral Strengths and Difficulties
eTable 3. Comparison of Norm Data Proportions to Simulated Proportions
eAppendix.

This supplementary material has been provided by the authors to give readers additional information about their work.

eFigure. Flow Chart

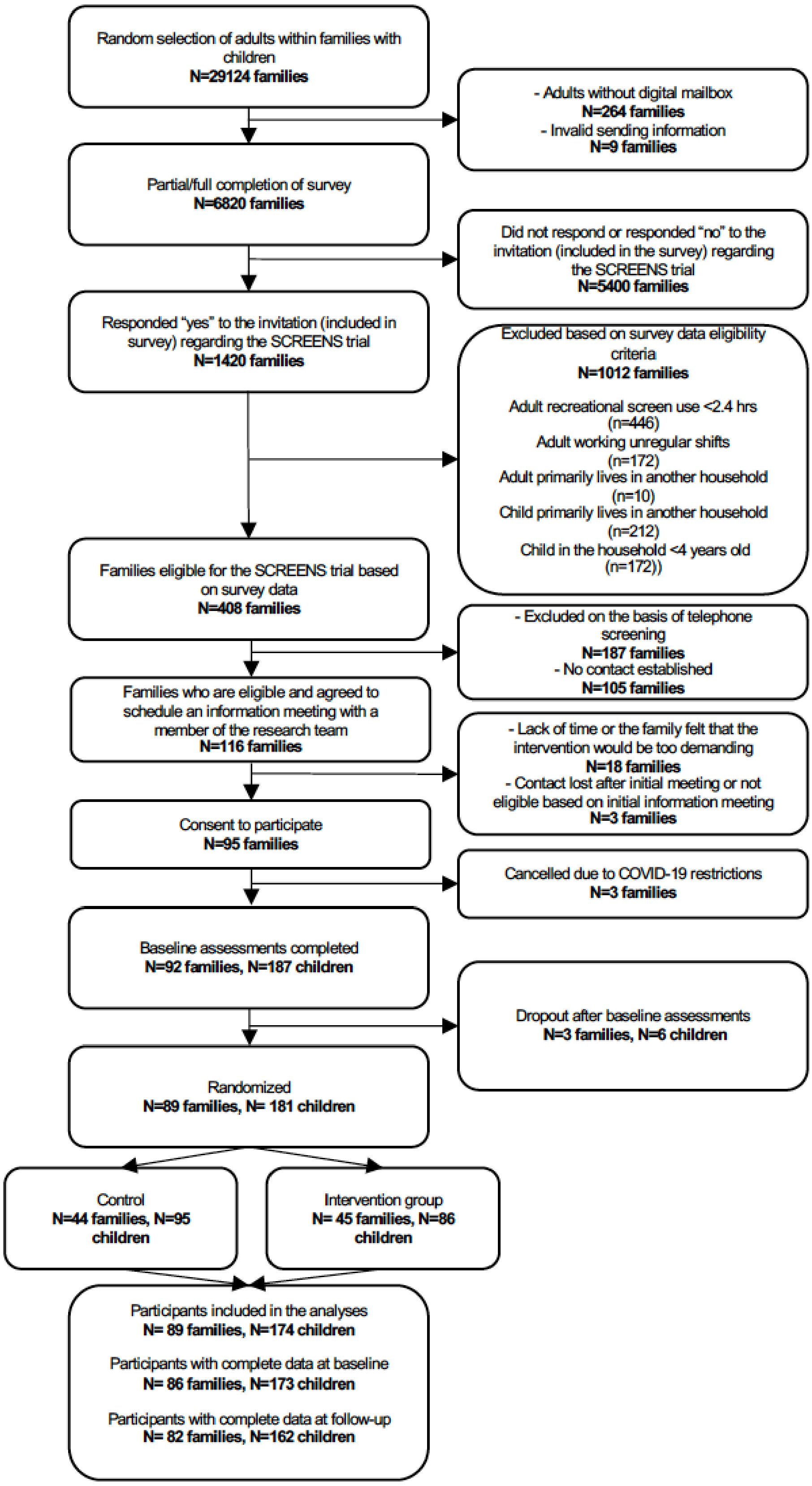

eFigure 1: Flow of participants from invitation to analysis

© 2024 Schmidt-Persson J et al. *JAMA Network Open.*

eTable 1. Comparison of Total Difficulties Scores at Baseline

	**Participants with complete data at baseline and follow-up (n=161)**	**Missing data at follow-up (n=12)***
**Age, years** mean (sd)	9.1 (2.6)	9.3 (2.6)
**Sex, females** n (%)	85 (52.8)	8 (66.7)
**Total screen time at baseline, hours/week** median (Q1-Q3)	16.0 (6.6-29.6)	19.4 (6.1-30.7)
**Total difficulties at baseline, points** median (Q1-Q3)	7 (4-10)	8 (5.5-13.5)

eTable 1: Median and quartiles are compared for participants with complete data at baseline and follow-up and for participants with missing data at follow-up. *Although 19 participants had missing data at follow-up only 12 of these participants had complete baseline data for the total difficulties score.

© 2024 Schmidt-Persson J et al. *JAMA Network Open.*

eTable 2. Analyses for Behavioral Strengths and Difficulties

**Outcomes**	**Control (n=91)**				**Intervention (n=82)**				**Mean difference in change**		
	Baseline	Change			Baseline	Change			Interaction		
	Mean (SD)	Mean (SD)	B1(c1-c0)	CI	Mean (SD)	Mean (SD)	B1(i1-i0)	CI	B3(t.e.)	CI	P-value
**Total difficulties score**	7.03 (4.54)	-0.06 (3.06)	-0.12	-0.81 to 0.58	7.29 (4.36)	-1.74 (3.21)	-1.74	-2.44 to -1.04	-1.67	-2.68 to -0.67	**0.001**
**Internalizing and externalizing symptoms**											
**Internalizing score**	3.21 (2.87)	0.11 (2.60)	0.02	-0.49 to 0.53	3.26 (3.03)	-1.03 (2.13)	-1.01	-1.54 to -0.48	-1.03	-1.76 to -0.29	**0.006**
**Externalizing score**	3.82 (2.88)	-0.17 (1.87)	-0.19	-0.62 to 0.23	4.04 (2.80)	-0.71 (2.16)	-0.73	-1.18 to -0.28	-0.54	-1.15 to 0.08	0.09

eTable 2: Baseline columns present raw mean and standard deviations. Change columns present estimated within-group mean change scores and 95% confidence intervals predicted from the regression models. Mean difference in change represents the estimated intervention effect (interaction between group allocation and time adjusted for age) predicted from the mixed tobit regression models.

© 2024 Schmidt-Persson J et al. *JAMA Network Open.*

eTable 2. Analyses for Behavioral Strengths and Difficulties (continued)

Outcomes	**Control (n=91)**				**Intervention (n=82)**				**Mean difference in change**		
	Baseline	Change			Baseline	Change			Interaction		
**SDQ subscales**											
**Conduct problems**	1.30 (1.43)	-0.14 (0.99)	-0.18	-0.40 to 0.05	1.24 (1.40)	-0.34 (1.13)	-0.33	-0.57 to -0.10	-0.15	-0.48 to 0.17	0.36
**Hyperactivity/inattention**	2.53 (2.05)	-0.02 (1.41)	-0.03	-0.35 to 0.30	2.79 (2.04)	-0.38 (1.68)	-0.40	-0.74 to -0.06	-0.37	-0.84 to 0.10	0.13
**Emotional problems**	2.38 (2.25)	-0.23 (2.18)	-0.31	-0.73 to 0.11	2.48 (2.25)	-0.81 (1.76)	-0.77	-1.22 to -0.33	-0.46	-1.08 to 0.15	0.14
**Peer problems**	0.82 (1.08)	0.33 (1.11)	0.31	0.10 to 0.52	0.78 (1.24)	-0.22 (0.85)	-0.23	-0.45 to -0.01	-0.54	-0.85 to -0.24	**0.001**
**Prosocial behavior**	8.49 (1.77)	-0.62 (1.58)	-0.64	-0.95 to -0.32	8.60 (1.40)	0.23 (1.37)	0.21	-0.12 to 0.54	0.84	0.39 to 1.30	**<0.001**

eTable 2: Baseline columns present raw mean and standard deviations. Change columns present estimated within-group mean change scores and 95% confidence intervals predicted from the regression models. Mean difference in change represents the estimated intervention effect (interaction between group allocation and time adjusted for age) predicted from the mixed tobit regression models.

© 2024 Schmidt-Persson J et al. *JAMA Network Open.*

eTable 3. Comparison of Norm Data Proportions to Simulated Proportions

	**Boys**		**Girls**	
	Norm data	After simulation	Norm data	After simulation
**Normal, %**	83.1	89.3	80.7	78.0
**Borderline, %**	7.1	7.2	9.2	14.0
**Abnormal, %**	9.8	3.5	10.1	8.0

eTable 3: The table present the proportions in each diagnostic category from the Danish norm data, and the estimated proportions in each diagnostic category after simulation of a widespread application of the screen media use reduction intervention. For more information see the supplemental text on the next page.

© 2024 Schmidt-Persson J et al. *JAMA Network Open.*

eAppendix.

Estimating the expected impact of reducing screen media use on the distribution of SDQ (Strengths and Difficulties Questionnaire) total difficulties score classifications in normal, borderline, and abnormal ranges in Danish normative data in 10-12-year-old children

The clinical relevance of the effect size (mean difference in change of -1.67 points in the total group and -2.52 in boys and -0.92 in girls between intervention and control) with respect to population impact can be quantitatively gauged by comparing intervention effects against normative data shifts in a defined population. We analyzed the expected impact of reducing screen media use on the SDQ total difficulties score among Danish children aged 10-12 years using normative Danish data, should a similar intervention be widely implemented. We simulated the expected distributional shifts in the SDQ total difficulties score resulting from the mean reductions. The norm data mean SDQ total difficulty scores are 6.02 points (SD 5.24) for boys and 5.27 points (SD 4.72) for girls. Distribution and cut-off scores in the norm data delineating normal, borderline, and abnormal score ranges are:

Boys:
Normal: 0-10 points, accounting for 83.1% of the population.
Borderline: 11-13 points, accounting for 7.1% of the population.
Abnormal: 14-40 points, accounting for 9.8% of the population.

Girls:
Normal: 0-8 points, accounting for 80.7% of the population.
Borderline: 9-11 points, accounting for 9.2% of the population.
Abnormal: 12-40 points, accounting for 10.1% of the population.

Normative SDQ Data from Denmark were obtained from URL: https://www.sdqinfo.org/norms/DanishNorms.html (accessed April 23 2024)) with the material described in Niclasen J, Teasdale TW, Andersen A-MN, et al. Psychometric properties of the Danish strength and difficulties questionnaire: the SDQ assessed for more than 70,000 raters in four different cohorts. PLoS ONE. 2012;7(2):e32025.

Adjustment of mean scores and estimated distributional change:
For boys: Mean after = 6.02 - 2.52 = 3.50 points
For girls: Mean after = 5.27 - 0.92 = 4.35 points

Using z-scores and the standard normal distribution's cumulative density function (CDF), we recalculated the expected proportions within each SDQ category post-intervention. These calculations were derived from the following z-score transformation:

$$z = \frac{(X - Mean)}{SD}$$

Where X is the cut-off score for the respective SDQ total difficulties range scores, *Mean* is the adjusted mean post-intervention, and *SD* is the standard deviation (assumed to the unaffected).

And the cumulative proportion calculations:

$$P\ (X \leq x) = \Phi\ (z)$$

Where Φ denotes the CDF of the standard normal distribution, $P\ (X \leq x)$ represents the probability that the total difficulties score X for a randomly selected child does not exceed the cut-off value x, where x corresponds to the upper limit of a specified SDQ category (e.g., 10 for the 'Normal' range in boys, and 8 for the Normal' range in girls), and z is the z-score calculated using the adjusted mean and standard deviation for that cut-off value.

© 2024 Schmidt-Persson J et al. *JAMA Network Open.*

The expected impact of reducing screen media use on the SDQ total difficulties score

This simulated analysis suggests that widespread application of similar interventions could result in a meaningful change in the proportions of children rated as 'abnormal' and 'borderline'.

For the Normal category cut-off (0-10 for boys, 0-8 for girls) we found the new proportions after the intervention to be approximately 89.3% for boys and 78.0% for girls.

For the Borderline category (11-13 for boys, 9-11 for girls), the proportions were estimated at 7.2% for boys and 14.0% for girls.

For the Abnormal category (14-40), we estimated 3.5% for boys and 8.0% for girls.

© 2024 Schmidt-Persson J et al. *JAMA Network Open.*
