# Supplementary material for: Screen Media Use and Mental Health of Children and Adolescents: A Secondary Analysis of a Randomized Clinical Trial
Source: JAMA Netw Open. 2024 Jul 12;7(7):e2419881. doi: 10.1001/jamanetworkopen.2024.19881 (PMC11245724; doi:10.1001/jamanetworkopen.2024.19881)
Supplement: Supplement 3. — Data Sharing Statement [file jamanetwopen-e2419881-s003.pdf]

Data Sharing Statement

Schmidt-Persson. Screen Media Use and Mental Health of Children and Adolescents. *JAMA Netw Open*. Published July 12, 2024. doi:10.1001/jamanetworkopen.2024.19881

Data
Data available: Yes
Data types: Other (please specify)
Additional Information: Deidentified data will be made available to other researchers upon request.
How to access data: Upon request to the corresponding author (jesp@kp.dk).
When available: With publication

Supporting Documents
Document types: Other (please specify)
Additional Information: Analytic code used for analyses will be shared upon request to the corresponding author (jesp@kp.dk).
How to access documents: Analytic code used for analyses will be shared upon request to the corresponding author (jesp@kp.dk).
When available: With publication

Additional Information
Who can access the data: Researchers whose proposed use of the data has been approved.
Types of analyses: Replication or exploratory analyses for researchers whose proposed use of the data has been approved.
Mechanisms of data availability: Upon request to the corresponding author (jesp@kp.dk).
